# Exploring Habitat Preferences, Suitability, and Illegal Trade Routes of Indian Pangolins in Western Pakistan: Implications for Conservation

**DOI:** 10.1002/ece3.72610

**Published:** 2026-01-09

**Authors:** Tariq Ahmad, Arshad Ali, Muhammad Farooq, Bo Li, Sayantani M. Basak, Tika Ram Poudel, Khuzin Dinislam

**Affiliations:** ^1^ College of Wildlife and Protected Area Northeast Forestry University Harbin China; ^2^ State Forestry and Grassland Administration Detecting Center of Wildlife Harbin China; ^3^ Department of Zoology Malakand University Chakdara Khyber Pakhtunkhwa Pakistan; ^4^ Department of Zoology, Wildlife and Fisheries PMAS‐Arid Agriculture University Rawalpindi Pakistan; ^5^ School of Biological, Earth and Environmental Sciences University College Cork Cork Ireland; ^6^ Feline Research Center of National Forestry and Grassland Administration, College of Wildlife and Protected Area Northeast Forestry University Harbin China; ^7^ Bashkir State Medical University Ufa Russia

**Keywords:** conservation, ensemble modeling, habitat preferences, habitat suitability, illegal poaching, Indian pangolin, MaxEnt, Random Forest, Support Vector Machine

## Abstract

The Indian pangolin ( *Manis crassicaudata* ; Manidae, Pholidota), a species categorized as “Endangered” on the IUCN Red List, is one of nine extant pangolin species in Asia. This study investigated habitat preference, habitat suitability, and illegal trade routes of the Indian pangolin in Pakistan's Khyber Pakhtunkhwa province. Habitat preference was determined by analyzing the distribution and density of pangolin signs across various land cover types. Habitat suitability for the species was assessed using the MaxEnt modeling approach and field data. Trade routes were identified using information from hunters, poachers, dealers, and local communities to understand the threats related to illegal wildlife trafficking. Results indicated significant differences in burrow distributions across habitats (*χ* ^2^ = 17.756, df = 6, *p* < 0.01), which suggest ecological preferences and adaptations. We complemented MaxEnt with Random Forest and Support Vector Machine models trained with the same predictors and spatial folds to validate robustness and characterize non‐linear effects. Across held‐out folds, SVM performed best, with RF and MaxEnt yielding comparable but lower discrimination; a TSS‐weighted ensemble provided a stable consensus SVM (mean AUC ≈ 0.61; TSS ≈ 0.33). Variable‐importance and partial‐dependence analyses consistently highlighted Elevation, NDMI, and NDWI as influential predictors. Several routes used for the illegal trade of Indian pangolin scales and whole animals were identified. The study also highlights the ongoing issues of illegal poaching and habitat intrusion, worsened by low local awareness and inadequate enforcement. The findings support a comprehensive conservation strategy that includes strict enforcement of wildlife protection laws, increased penalties for poaching, community‐based monitoring through targeted awareness campaigns, local wildlife patrols, and ongoing scientific research to support habitat restoration, adaptive management, and evidence‐based policy development. Community‐based conservation initiatives and improved wildlife law enforcement at key trafficking hubs could significantly reduce poaching pressure.

## Graphical abstract

This study assessed habitat preferences, suitability, and illegal trade routes of the endangered Indian pangolin in Pakistan's Khyber Pakhtunkhwa province. Using field surveys and MaxEnt modeling, we identified fragmented habitats with only 22% classified as highly suitable, alongside key poaching and trafficking pathways. Findings emphasize the need for integrated conservation efforts combining habitat protection, community engagement, and stronger law enforcement.

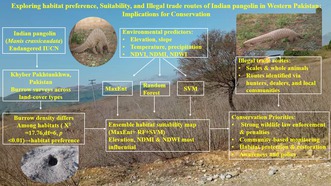

## 1 Introduction

Indian pangolin ( *Manis crassicaudata* ; Manidae, Pholidota) is one of the nine extant species of pangolin that are found in Asia, categorized as “Endangered” by the IUCN Red List of Threatened Species (Gu et al. 2023; Mahmood et al. 2019). Native to South Asia, it inhabits countries such as Pakistan, Sri Lanka, India, and Southern Nepal (Ahmad and Li 2024). Within Pakistan, the Indian pangolin has been reported across various regions of all four provinces, as well as select districts within Azad Jammu and Kashmir (Akrim et al. 2017; Roberts 1997), typically residing in subtropical thorn forests and barren hilly terrains (Roberts 1997), encompassing habitats from grasslands to moist, dry, and thorn forests (Pai 2008). The species occurs at naturally low population densities and prefers forested surroundings of various types (Gaudin et al. 2006). It can also be found in abandoned wasteland close to populated areas (Yang et al. 2007). The species inhabits a variety of settings in Northern Khyber Pakhtunkhwa, Pakistan, including forests, grasslands, and agricultural edges, demonstrating outstanding adaptation to a wide range of ecological niches (Roberts 1997). According to the research, *M. crassicaudata* may adapt to a range of habitats throughout its geographic range (Chakkaravarthy 2012). Habitat features such as vegetation cover, tree species composition, and geological features (such as the presence of water sources, rock boulders, and soil characteristics) have been recognized as important factors worth considering in the characterization of burrowing habitats of pangolins (Mahmood et al. 2015; Pabasara et al. 2015). In addition, Indian pangolins prefer natural forests over agricultural land and human localities (Mahmood et al. 2021). Vegetation investigation of the habitat of the Indian pangolin indicated that pangolin burrows were associated with a specific tree and shrub species, including *Zizyphus nummularia*, *Acacia nilotica* , and *Ziziphus mauritiana* , showing a species preference for certain types of vegetation in its habitat (Mahmood et al. 2015).

All pangolin species (African and Asian) are on appendix I of CITES (2017) and are considered to be the most trafficked wild mammal internationally (Challender et al. 2014), facing significant threats from illegal wildlife trade across both Asia and Africa (Challender and O'Criodain 2020). This trade is documented as having a severe influence on the status of pangolin populations (Challender et al. 2014). However, the Indian pangolin faces major threats from habitat destruction and growing illegal wildlife trafficking, fueled by demand for its meat and scales in traditional medicine and exotic cuisine markets throughout Asia (Challender et al. 2015; Challender and Hywood 2011). Recent research has found catastrophic declines in pangolin populations as a result of widespread poaching and trafficking, which are exacerbated by poor enforcement of wildlife protection legislation in countries such as Pakistan; 179 Indian pangolins were killed in Khyber Pakhtunkhwa (Peshawar, Mardan, Nowshera, Kohat) and 59 from Punjab (Chakwal, Jhelum, Attock, Rawalpindi) (Ahmad and Li 2024; Waseem et al. 2020). The IUCN estimates show a decrease of 50% in the global Indian pangolin population over the next 21 years, emphasizing the need to protect the species against its illegal trade (Mahmood et al. 2021).

The distribution of the Indian pangolin is influenced by temperature, precipitation, elevation, ants and termites, human settlements, land cover, and other factors (Mahmood et al. 2015; Suwal et al. 2022). Furthermore, comprehending the intricate interplay between habitat selection and human‐caused changes is crucial. Land use changes, urbanization, and agricultural activities all have a significant impact on the Indian pangolin's habitat and survival (Shilereyo et al. 2021). Pangolins usually avoid heavily modified landscapes; although some species, such as the Chinese pangolin in Nepal, can adapt to agricultural areas with sufficient food resources and low human disturbance (Karawita et al. 2018; Wilske et al. 2015).

This complex situation emphasizes the importance of establishing buffer zones and executing tight anti‐poaching measures in critical ecosystems. The conservation of the Indian pangolin in Pakistan demands a multifaceted strategy that includes stringent law enforcement, habitat protection, and community engagement. This study explores habitat preferences, suitability, and trade routes of the Indian pangolin in Western Pakistan to report critical knowledge gaps about its distribution and threats in this region. We assume that some ecological factors, including vegetation cover, water availability, and human disturbance, have a major impact on habitat suitability in Western Pakistan. Furthermore, the study aims to identify the trade routes contributing to pangolin trafficking and also provide data‐driven insights for targeted conservation measures. By focusing on Western Pakistan, this research lays the basis for region‐specific conservation planning, addressing anthropogenic drivers and ecological factors of pangolin decline.

## 2 Materials and Methods

### 2.1 Study Area

**FIGURE 1:**
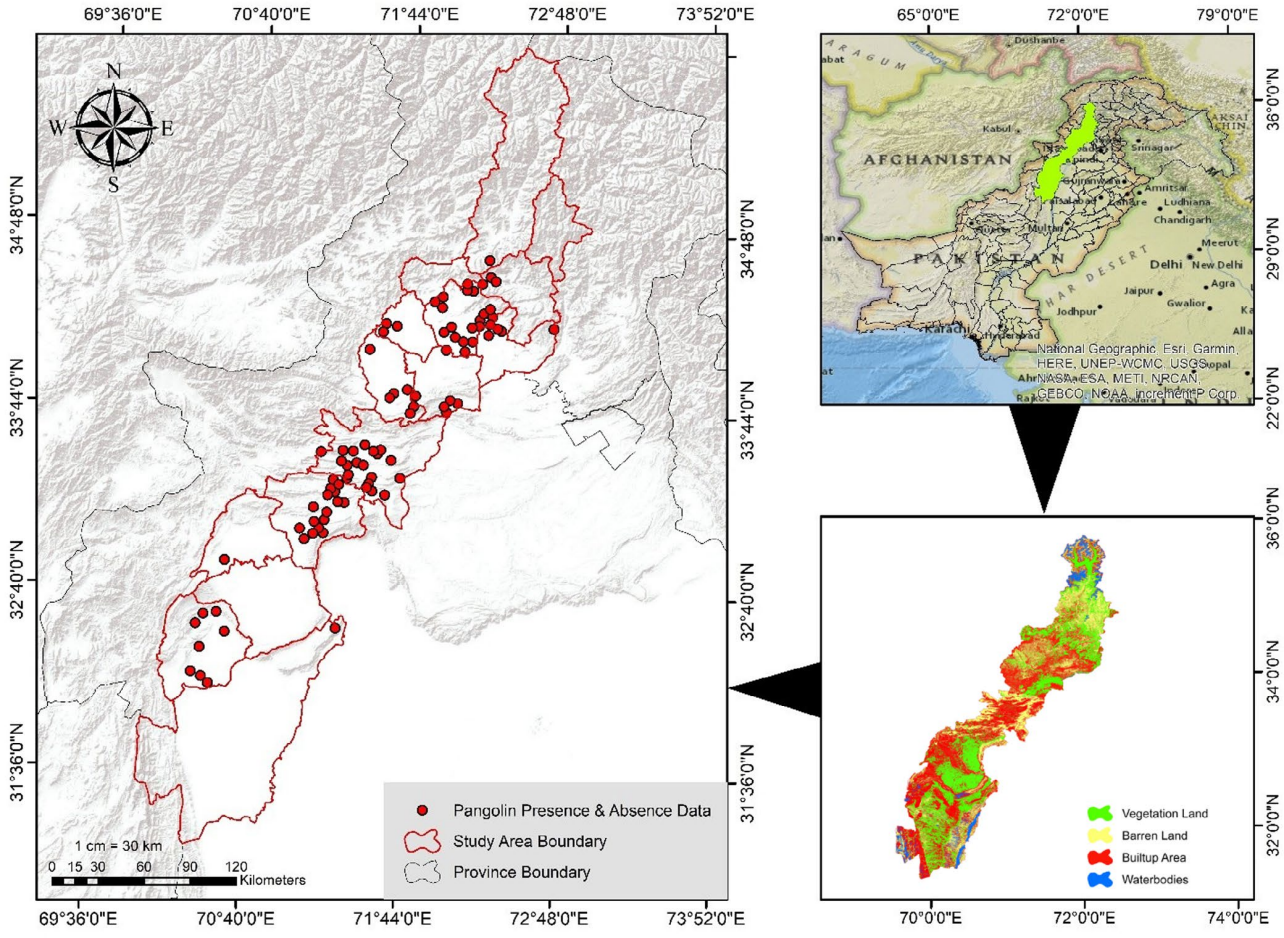
Geographic distribution map of study areas in Khyber Pakhtunkhwa (KP) highlighting areas with recorded sightings and evidence of Indian pangolins, along with land cover map.

The current study was undertaken in 15 districts of the Khyber Pakhtunkhwa (KP) province of Pakistan. KP's climate varies greatly, from dry places to some of Pakistan's wettest locations during the monsoon season (mid‐June to mid‐September). The province is predominantly mountainous, resulting in wide daily and annual temperature fluctuations (Ahmad et al. 2022). Khyber Pakhtunkhwa, often known as KP or KPK, is the third‐largest province in Pakistan, with a total human population of 40.85 million, and the fourth largest by geographical area at 101,741 km^2^ (Hussan et al. 2024). The research area spans 15 districts and 40,183 km^2^. From 1998 to 2017 (19 years), the province's human population increased from 17.7 to 30.5 million, reaching 40.85 million in 2023 (Pakistan Bureau of Statistics 2023). KP is located at 34.9526° N, 72.3311° E, with a diverse landscape that includes rocky mountain ranges, valleys, plains bordered by hills, undulating submontane zones, and dense agricultural farms. The KP province shares borders with Gilgit‐Baltistan to the northeast, the Azad Jammu and Kashmir region to the east, Balochistan to the south, and Afghanistan to the west (Figure 1). The average elevation is 2135 m (a.s.l.), with minimum and maximum elevations of 170 and 7856 m, respectively (Hussan et al. 2024). The climate varies greatly throughout the research area. Peshawar has a hot semi‐arid climate with summer temperatures surpassing 40°C and an average annual precipitation of 400 mm. Dera Ismail Khan, located in the southwest of the study area, experiences harsher summers with temperatures exceeding 45°C and reduced humidity (Khan et al. 2010). Swat, in the northwest, has a temperate continental climate with cold, snowy winters and pleasant summers, with an annual rainfall of 600 mm (Ali et al. 2022). KP is home to a diverse animal population, including the snow leopard ( *Panthera uncia* ), common leopard ( *Panthera pardus* ), Himalayan lynx ( *Lynx lynx isabellinus* ), Kashmir markhor (*Capra falconeri cashmiriensis*), Himalayan ibex ( *Capra sibirica* ), urial ( *Ovis vignei* ), and Indian pangolin ( *Manis crassicaudata* ) (Ahmad et al. 2025). The province has witnessed a substantial decline in wildlife, with 44 species listed as critically endangered or near‐threatened (Ahmad et al. 2024; Khattak et al. 2021). The dominant plant species are *Acacia modesta* (Phulai), *Monotheca buxifolia* (Gurgurah), *Vachellia nilotica* (Kikar), *Olea ferruginea* (Olive, Zaithoon), *Zizyphus mauritiana* (Ber), and *Dodonaea viscosa* (*Sanatha*, *Ghwarahsky*). Several of these species, particularly Zizyphus mauritiana and Acacia modesta, are associated with high pangolin activity due to their dense cover and abundant ant and termite populations, which provide vital foraging habitat (Khattak et al. 2021; Rahman et al. 2024).

### 2.2 Study Design

From January 2021 to June 2023, a total of 302 field visits were conducted across 53 sites in 15 districts. Each site was revisited approximately every 6 months to ensure comprehensive data collection for habitat preferences and habitat suitability modeling. Information about trade routes was gathered through interviews with law enforcement officers, hunters who were in prison, local communities, and market surveys, identifying major trafficking corridors and assessing threats. Detailed methodology related to habitat preference, habitat suitability modeling, and illegal trade of the Indian pangolin is given below.

### 2.3 Habitat Preference

**FIGURE 2:**
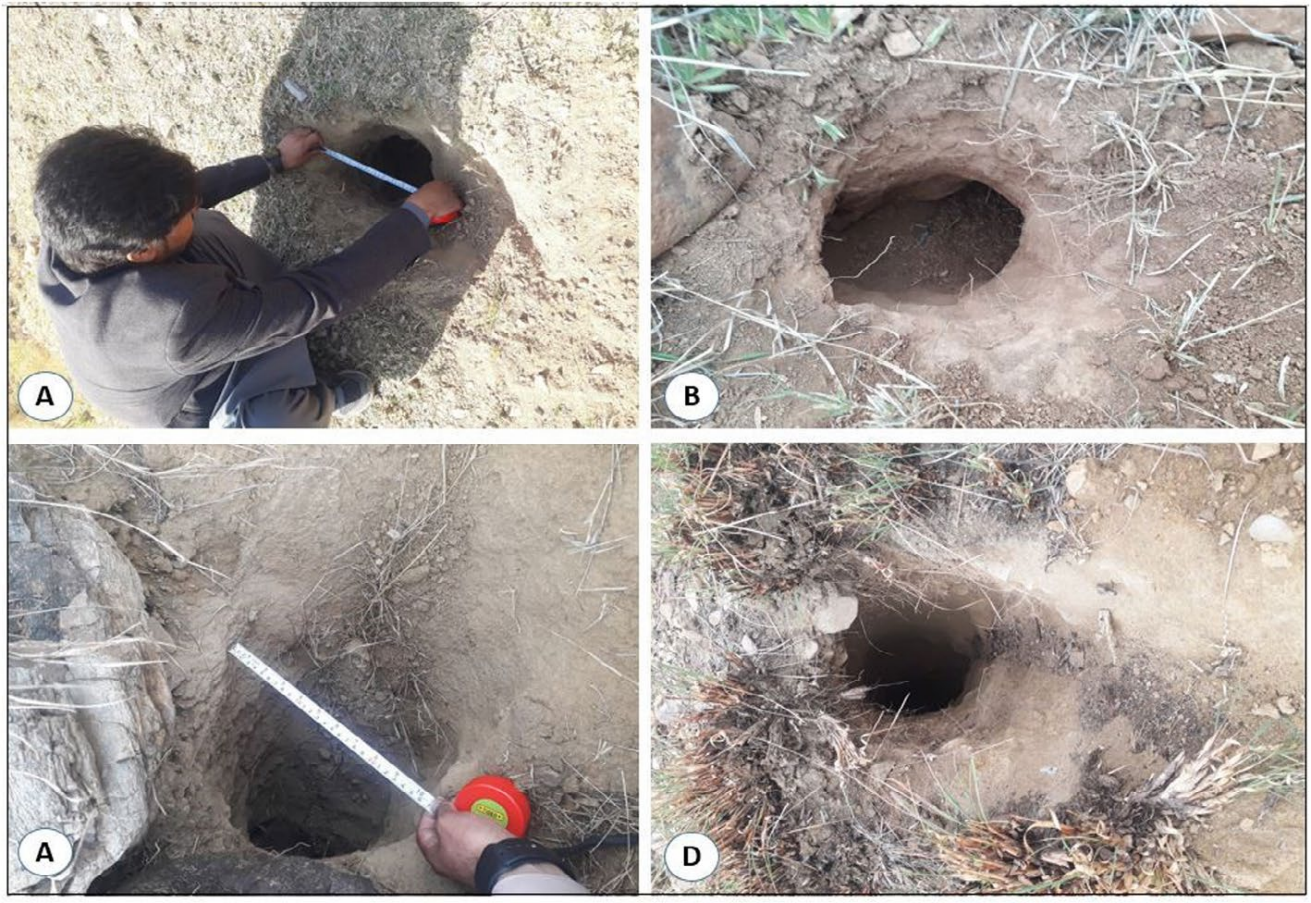
Methods of identifying active pangolin burrows: (A) measuring burrow entrance; (B–D) examples of active burrow entrances.

To study the habitat preference, direct (captures, sightings, dead bodies) and indirect signs (feces, burrows) of the Indian pangolin were recorded during field surveys. A fixed‐width line transect measuring 70 m long and 10 m wide (19.6 ha) was used for data collection. Transect locations were selected based on an organized assessment of the study area's environmental gradient, including factors such as vegetation type, elevation, and soil composition. We prioritized areas where pangolin activity was previously detected or where habitat features, such as loose sandy or loamy soils and dense vegetation, were most suitable for burrow formation (Mahmood et al. 2014). To avoid overlapping sample regions, adjacent transects were kept at a minimum distance of 300 m apart. The sample size was based on a preconceived design, confirming the representation of the three habitat types in proportion to their relative areas in the study region. A total of 280 transects were set across three habitat types: 95 on forest land, 100 on grassland, and 85 on agricultural land. While agricultural landscapes are normally considered modified, the agricultural areas in this study are semi‐modified, consisting of small‐scale farms with surrounding natural patches and vegetation that still provide suitable habitat for pangolins. The burrows were identified based on unique characteristics, including shape, size, and entrance width. These burrows were typically found in areas with loose, loamy soils or sandy substrates, often in grasslands, forests, or submontane zones (Figure 2). A group of five experts, comprising local community members, wildlife staff, and former hunters/poachers, was consulted to validate burrow identification. They were trained using standardized guidelines and provided input based on key markers, such as fecal samples, claw marks, footprints, scratch marks, and burrow morphology. Burrow confirmation relied on these specific characteristics, with quantitative assessments ensuring consistency (Karawita et al. 2018). Geographic locations of pangolin and indirect signs were used to create a distribution map.

Soil types were measured using laboratory analysis. Topographical features, such as elevation, were measured using GPS devices with built‐in altimeters, while slope was measured through a clinometer. These topographical factors were considered important because they influence water drainage, vegetation types, and the ease with which pangolins can dig burrows. Other key environmental variables, including termite and ant colonies and soil types, were documented, as these factors significantly influence pangolin habitats (Akrim et al. 2017; Mahmood et al. 2015).

For vegetation analysis, we employed a stratified random sampling design in different types of habitats (forest, agricultural land, and grassland), dividing the study area into plots with a 100‐m radius. Within these plots, the quadrat method was employed for vegetation analysis, utilizing quadrats of varying sizes: 10 m × 10 m for the trees, 3 m × 3 m for shrubs, and 1 m × 1 m for herbaceous species. Plant species lists were compiled during field surveys. The following formulas were used to calculate density, relative density, relative frequency, relative cover, and the importance value index (IVI).

$$\text{Density}=\frac{\text{Total number of individuals of}\;a\;\text{species in}\;\mathrm{all}\;\text{quadrants}}{\text{total number of quadrants studied}}$$

$$\text{Relative Density}=\frac{\text{Density of}\;a\;\text{species}\;A}{\text{Total density of}\;\mathrm{all}\;\text{species}}\times 100$$

$$\text{Relative Frequency}=\frac{\text{Density of}\;a\;\text{species}\;A}{\text{Total frequency of}\;\mathrm{all}\;\text{species}}\times 100$$

$$\text{Relative Cover}=\frac{\text{Total basal cover species}\;A}{\text{Total basal cover of}\;\mathrm{all}\;\text{species}}\times 100$$

IVI=Density+Relative Density+Relative Frequency+Relative Cover

#### 2.3.1 Statistical Analysis

The R program version (4.3.2) (R Development Core Team 2010) was used for inferential statistics, with chi‐squared tests revealing connections between categorical variables such as land cover types (e.g., forest, grassland, agricultural land) and the pangolin signs (e.g., burrows, tracks). This test assessed whether the distribution of pangolin signs was associated with different land cover types in the study area. The chi‐square goodness‐of‐fit (GOF) test was also applied to evaluate if observed frequencies of pangolin signs deviated significantly from expected distributions under the null hypothesis. All pangolin sightings used in this analysis were based on direct and indirect field signs recorded during the surveys.

#### 2.3.2 Spatial Analysis With ArcGIS

**FIGURE 3:**
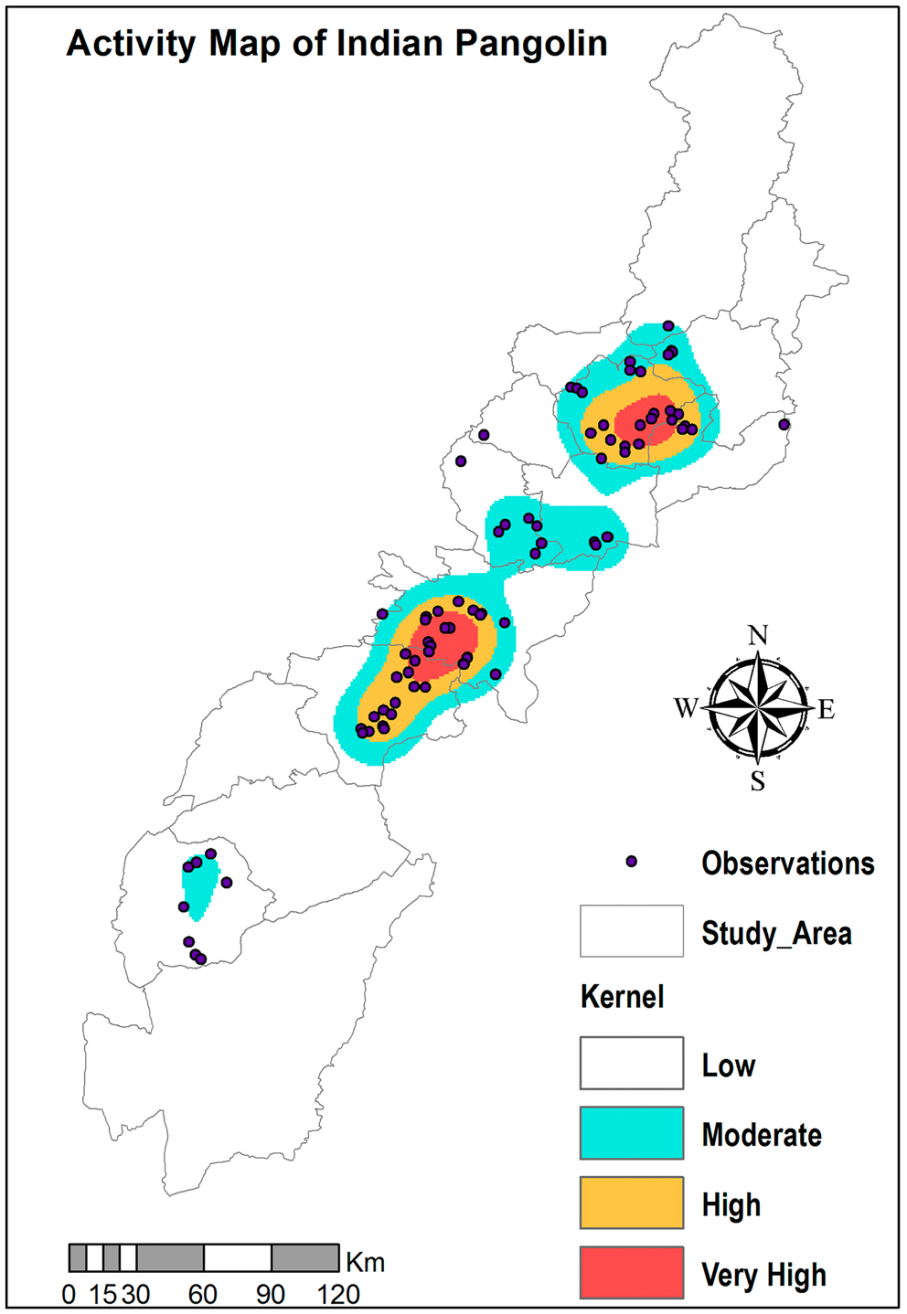
Kernel density map showing the activity of Indian pangolin in western Pakistan.

Spatial analyses were carried out in ArcGIS (ESRI 2011) to visualize patterns in the distribution and density of pangolin sightings in Khyber Pakhtunkhwa. Heat maps were generated to represent areas of high pangolin activity, highlighting regions that are particularly important for conservation. These spatial tools evaluate the spatial clustering of pangolin signs. This allowed us to better understand how the landscape and environmental features interact with the species' distribution (Figure 3).

### 2.4 Habitat Suitability Analysis

MaxEnt (Maximum Entropy) modeling, a popular method for species habitat suitability modeling, was conducted in the following steps:

- Data acquisition and preparation: To assess the habitat suitability of the Indian pangolin, the study area was divided into 10 km × 10 km grid cells GPS location of each occurrence point was recorded. Biological variables were downloaded from the Worldclim website (http://www.worldclim.org/). Normalized difference vegetation index (NDVI) was derived for the Landsat 8 images (https://earthexplorer.usgs.gov/). Digital Elevation model (DEM) was retrieved from the USGS Earth Explorer database (https://earthexplorer.usgs.gov/). Aspect and slope were derived from the DEM by using “Surface Tools” in ArcGIS. Water bodies data (shape files) were downloaded from the DIVA GIS website (https://www.diva‐gis.org/), and human settlements data were retrieved from the Humanitarian Data Exchange website (https://data.humdata.org/). The “Euclidean Distance” tool in ArcGIS calculates the straight‐line distance from water bodies and human settlements, converting these features into a raster format to align with the bioclimatic data (Table 1).
- Selection of environmental variables: We conducted a Pearson's correlation analysis on the bioclimatic variables to identify and exclude highly correlated pairs (*r* > 0.8), ensuring that only non‐collinear variables were incorporated into the MaxEnt model. Following this analysis, only two variables out of 19 were retained: Bio_01 (annual mean temperature) and Bio_12 (annual precipitation). The selection criteria of variables were based on ecological relevance to the Indian pangolin and correlation with species presence. Bio_01 was chosen due to its critical influence on pangolin distribution, as the species is sensitive to temperature extremes. Bio_12 was selected for its role in determining vegetation cover and food availability, thereby impacting habitat suitability.
  We incorporated proximity to water bodies and human settlements as predictor variables, calculated using Euclidean distance. The human settlements dataset enabled quantification of human encroachment, a critical factor influencing habitat suitability for the Indian pangolin. Water availability was also considered due to its essential role in supporting preferred vegetation types and pangolin survival. Additionally, we included the NDVI as a variable, quantifying vegetation health and productivity. NDVI values, ranging from −1 to +1, indicate vegetation greenness and vitality, with higher values corresponding to healthier vegetation and lower values indicating degraded or sparse vegetation. We relied on NDVI as a proxy indicator of vegetation cover and health, which is important for assessing habitat suitability for the Indian pangolin. Bias correction “bias_con” file was created by following the guidelines of Young et al. (2011).

**TABLE 1:** Bioclimatic and other environmental variables used in MaxEnt modeling for the Indian pangolin.

S. No	Variable	Source
1.	BIO1_Annual Mean Temperature	Worldclim website (http://www.worldclim.org/) (30 s)
2.	BIO2_Mean Diurnal Range (Mean of monthly (max temp—min temp))	Worldclim website (http://www.worldclim.org/) (30 s)
3.	BIO3_Isothermality (BIO2/BIO7) (×100)	Worldclim website (http://www.worldclim.org/) (30 s)
4.	BIO4_Temperature Seasonality (standard deviation × 100)	Worldclim website (http://www.worldclim.org/) (30 s)
5.	BIO4_Max Temperature of Warmest Month	Worldclim website (http://www.worldclim.org/) (30 s)
6.	BIO6_Min Temperature of Coldest Month	Worldclim website (http://www.worldclim.org/) (30 s)
7.	BIO7_Temperature Annual Range (BIO5‐BIO6)	Worldclim website (http://www.worldclim.org/) (30 s)
8.	BIO8_Mean Temperature of Wettest Quarter	Worldclim website (http://www.worldclim.org/) (30 s)
9.	BIO9_Mean Temperature of Driest Quarter	Worldclim website (http://www.worldclim.org/) (30 s)
10.	BIO10_Mean Temperature of Warmest Quarter	Worldclim website (http://www.worldclim.org/) (30 s)
11.	BIO11_Mean Temperature of Coldest Quarter	Worldclim website (http://www.worldclim.org/) (30 s)
12.	BIO12_Annual Precipitation	Worldclim website (http://www.worldclim.org/) (30 s)
13.	BIO13_Precipitation of Wettest Month	Worldclim website (http://www.worldclim.org/) (30 s)
14.	BIO14_Precipitation of Driest Month	Worldclim website (http://www.worldclim.org/) (30 s)
15.	BIO15_Precipitation Seasonality (Coefficient of Variation)	Worldclim website (http://www.worldclim.org/) (30 s)
16.	BIO16_Precipitation of Wettest Quarter	Worldclim website (http://www.worldclim.org/) (30 s)
17.	BIO17_Precipitation of Driest Quarter	Worldclim website (http://www.worldclim.org/) (30 s)
18.	BIO18_Precipitation of Warmest Quarter	Worldclim website (http://www.worldclim.org/) (30 s)
19.	BIO19_Precipitation of Coldest Quarter	Worldclim website (http://www.worldclim.org/) (30 s)
20.	Normalized Difference Vegetation Index (NDVI)	Derived from the Landsat 8 images which were downloaded from the GloVis‐USGS website (https://glovis.usgs.gov/) (30 m)
21.	DEM (Elevation)	Downloaded from website https://earthexplorer.usgs.gov/ (28 m)
22.	Aspect	Calculated from DEM (30 m)
23.	Slope	Calculated from DEM (30 m)
24.	Distance from Human Settlements	Human Settlements data was downloaded from the website (https://data.humdata.org/), and the Euclidian distance tool was used to calculate the distance
25.	Distance from the Water body	The water body was downloaded from the DIVA‐GIS website (https://www.divagis.org/). The Euclidian distance tool was used to calculate the distance

### 2.5 Comparative Modeling With Random Forests and SVM

To evaluate whether MaxEnt predictions were robust to algorithmic choice and to explore potential non‐linear effects and variable interactions, we implemented a comparative machine‐learning framework using Random Forests (RF) and Support Vector Machines (SVM). We used the same predictors and the same spatially explicit fivefold scheme already defined in our dataset (column fold). Models were trained on presence–background data with pseudo‐absences as described above. For each fold, models were fitted on four folds and evaluated on the held‐out fold; out‐of‐fold predictions were concatenated for performance estimation.

RF models were fitted with probability outputs (ranger; 800 trees; mtry ≈ √*p*; min node size = 5). SVM models used an RBF kernel with light regularization (cost = 1, *γ* = 1/*p*). MaxEnt models (maxnet) were re‐fit on the same folds using linear+quadratic features to prioritize stability. We computed AUC, TSS (threshold maximizing sensitivity + specificity − 1), and PR‐AUC for each fold and summarized means across folds (Figure 13; Table 3). We quantified variable importance using (i) RF impurity importance and (ii) permutation‐based AUC drop for SVM and MaxEnt. To visualize non‐linear responses, we produced partial‐dependence curves (PD) for the top RF predictors. Finally, we formed a simple TSS‐weighted ensemble of the three models using out‐of‐fold probabilities to summarize concordant signal (Table S1). All analyses were conducted in R (packages: maxnet, ranger, e1071, pROC, PRROC, pdp).

#### 2.5.1 Data Formatting

Each raster layer was projected to the World Geodetic System (WGS) 1984. Environmental layers were then extracted using the “Extract by Mask” tool in ArcGIS to ensure uniform extent, cell size (30 m × 30 m), and coordinate system, as required for MaxEnt analysis. The layers were subsequently converted to ASCII format using the “Raster to ASCII” conversion tool. Species occurrence records were spatially thinned to a single point per raster cell, minimizing spatial biases and ensuring data independence. Due to the absence of published data on the home range of the Indian pangolin, we used the Sunda pangolin's ( *Manis javanica* ) home range (~1.58 km^2^) as a proxy minimum distance threshold (Gray et al. 2023). All 115 occurrence points were for the Indian pangolin, obtained primarily from field observations in this study, supplemented with verified records from the published literature and the Wildlife Department. After applying spatial autocorrelation analysis, we retained 80 occurrence points for further analysis.

#### 2.5.2 MaxEnt Model Implementation

The habitat suitability modeling was carried out using MaxEnt software (version 3.4.4), which used environmental data to forecast suitable pangolin habitats. This software is widely recognized for its success in ecological modeling of species distribution, which uses a maximum entropy approach to forecast species distribution throughout a given area (Phillips et al. 2006). Presence records of Indian pangolin and environmental variables (in ASCII format) were input into the MaxEnt software to develop a habitat suitability model. Variables such as termite or ant and soil type abundance were excluded from the MaxEnt analysis because spatially continuous data for these factors were not available across the entire study area. However, they were examined separately in the field‐based habitat assessment. For MaxEnt model implementation, we used 80% (*n* = 64) of the occurrence points for training the model and 20% (*n* = 16) for testing the model's predictive accuracy, ensuring a robust evaluation of its generalizability. A bias correction file (“bias_con”) was also incorporated into MaxEnt to address sampling bias in species distribution modeling. We also used 5000 pseudo‐absence points (background points) in the modeling process, which were randomly selected from areas outside the known presence points. These pseudo‐absence points are essential for modeling species distribution, providing the algorithm with locations where the species is likely absent, which helps fine‐tune predictions. The jackknife procedure was implemented for the percentage contribution of each variable in determining the habitat suitability. We used 15 bootstrap iterations, and the output format was configured to produce logistic output.

#### 2.5.3 Model Evaluation

Model evaluation was done by using the area under the curve (AUC) of the receiver operating characteristic (ROC) curve (Elith et al. 2006). The software eased the formation of response curves for each environmental condition, providing a clearer understanding of how each variable affects pangolin habitat suitability. The software facilitated the creation of response curves for each environmental variable, providing insight into how each factor influences pangolin habitat suitability. The model's predictive accuracy was assessed via ROC curve analysis, a statistical method that evaluates sensitivity and specificity without relying on a fixed threshold. AUC values, ranging from 0.8 (good) to 1.0 (outstanding), provided a scalar measure of prediction accuracy (Ismaili et al. 2024; Pearce and Ferrier 2000). We also used true skill statistics (TSS) to assess the predictive power of our model. AUC_Diff_ value was also computed to check whether either model is overfitting or underfitting.

#### 2.5.4 Interpretation and Analysis

Model performance was evaluated using the AUC value. Variable importance was assessed via jackknife tests, and response curves illustrated relationships between predictors and habitat suitability. The habitat suitability index (HSI) map was reclassified into three categories: highly suitable, moderately suitable, and least suitable using the “Reclassify” tool in ArcGIS.

### 2.6 Public Perception and Trade Routes

**FIGURE 4:**
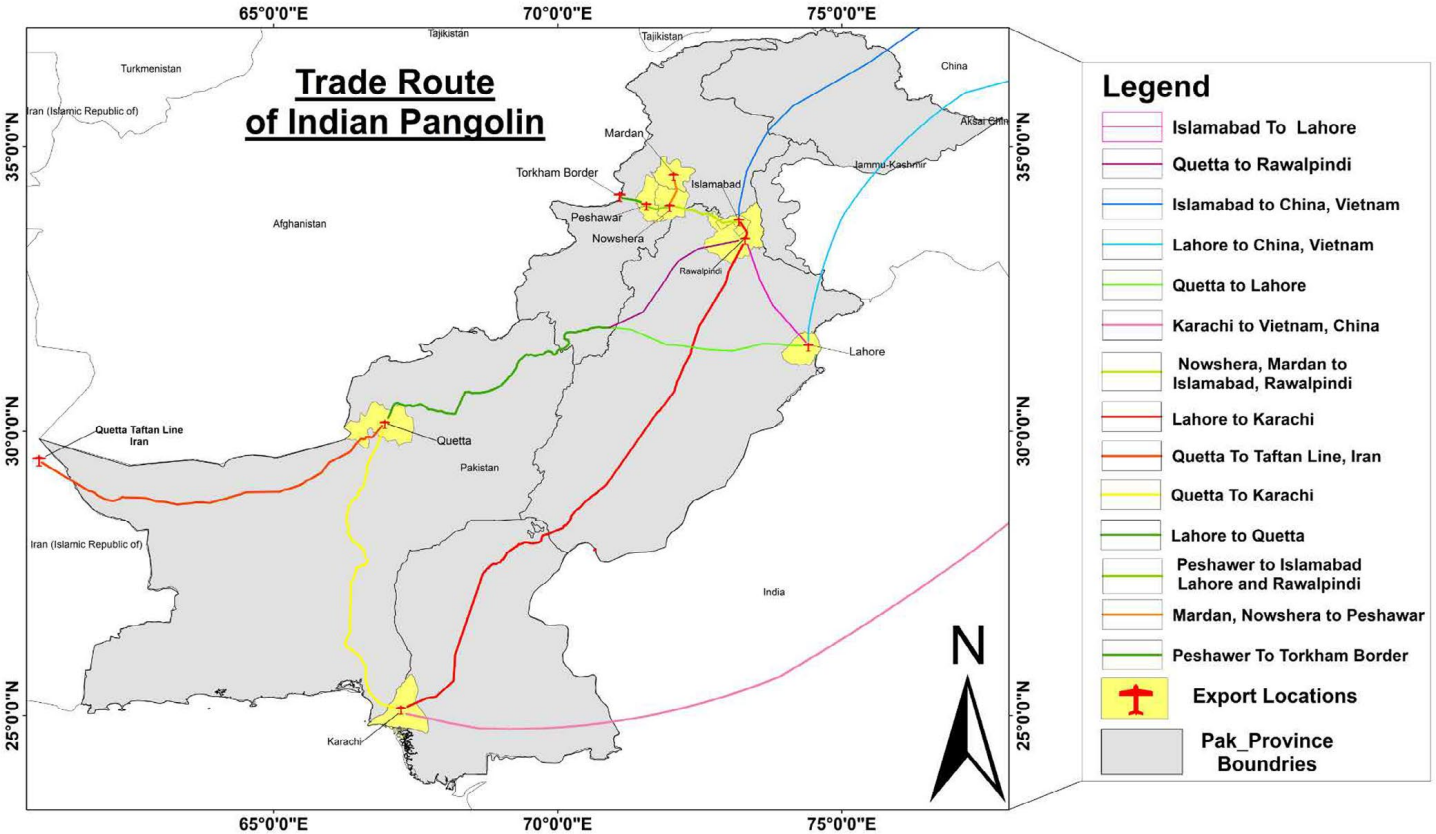
Map of Pakistan showing the major illegal trade routes of the Indian pangolin, illustrating the flow of trade from local markets to regional hubs.

To gain a better understanding of the socioeconomic dynamics impacting Indian pangolin conservation, we performed a comprehensive survey focusing on public perception of Indian pangolin conservation, illegal trafficking channels, associated threats, and community‐reported hunting practices. A total of 255 questionnaires and 196 interviews were conducted using a series of carefully designed questions covering pangolin sightings, trade routes, trafficking sources, capture methods, and awareness of protection laws. Questionnaires were completed by school students (*n* = 115), shopkeepers (*n* = 55), and local community members (*n* = 85), while interviews were conducted with law enforcement officers (*n* = 34), hunters in prison (*n* = 17), active hunters (*n* = 37), shepherds (*n* = 55), and local community members (*n* = 53). Some individuals participated in both questionnaires and interviews. Interviews were conducted verbally, while the questionnaires required written responses; this difference represented the primary methodological difference between the two approaches. All participants lived in or near pangolin habitats. The questionnaire was designed to provide nuanced insights into local groups' awareness, attitudes, and activities surrounding pangolin conservation. Questions were designed to elicit knowledge about both direct and indirect pressures on pangolin populations, such as hunting frequency, trade practices, and the perceived worth of pangolin‐related products in traditional and current usage. Major cities including Peshawar, Rawalpindi, Lahore, and Quetta were included in the study's geographic coverage because, despite their high levels of urbanization, they are either close to pangolin habitats or important centers for wildlife trade routes that have an impact on these populations (Figure 4). These locations were chosen based on preliminary data showing considerable wildlife trade activities that could influence pangolin populations. In partnership with local wildlife agencies and conservation NGOs, our team spoke with hunters and dealers in these marketplaces to acquire important information on pangolin trade routes, both within and across national boundaries.

Using ArcGIS 10.8.2, interview‐derived spatial data identifying key transit cities and border points was georeferenced and cross‐validated with law enforcement and conservation agency records to create maps of likely trade. This GIS‐based approach enabled the visualization of complex trading networks, resulting in a clear portrayal of the channels used to unlawfully transport pangolins. These maps help identify important intervention sites where conservation activities can be strategically targeted to effectively disrupt illegal wildlife trading chains. To explore habitat threats to Indian pangolins, we conducted surveys and interviews with nomads, local communities, hunters, college and university teachers, and wildlife officials. Data on habitat degradation factors, including logging, agriculture, urban expansion, deforestation, and grazing, were collected through direct field observations, structured interviews, and questionnaires. Field assessments complemented these data by providing direct evidence of habitat loss and pangolin‐killing practices. The incorporation of these approaches allowed us to identify both the methods and underlying motivations driving these threats, yielding critical insights into the conservation challenges impacting pangolin habitats.

### 2.7 OriginPro for Public Opinion Data

OriginPro 2024b (OriginLab Corporation 2024) also played an important part in evaluating public opinion survey data, computing percentages, and permitting the graphical display of community responses to pangolin conservation. The software helped calculate percentages of respondents supporting conservation efforts and visualize community responses. Bar charts and pie charts were used to display the results, while inferential statistics, such as confidence intervals, were computed to assess the reliability of the responses.

## 3 Results

### 3.1 Distribution of Burrow Types Across Habitat Types

**FIGURE 5:**
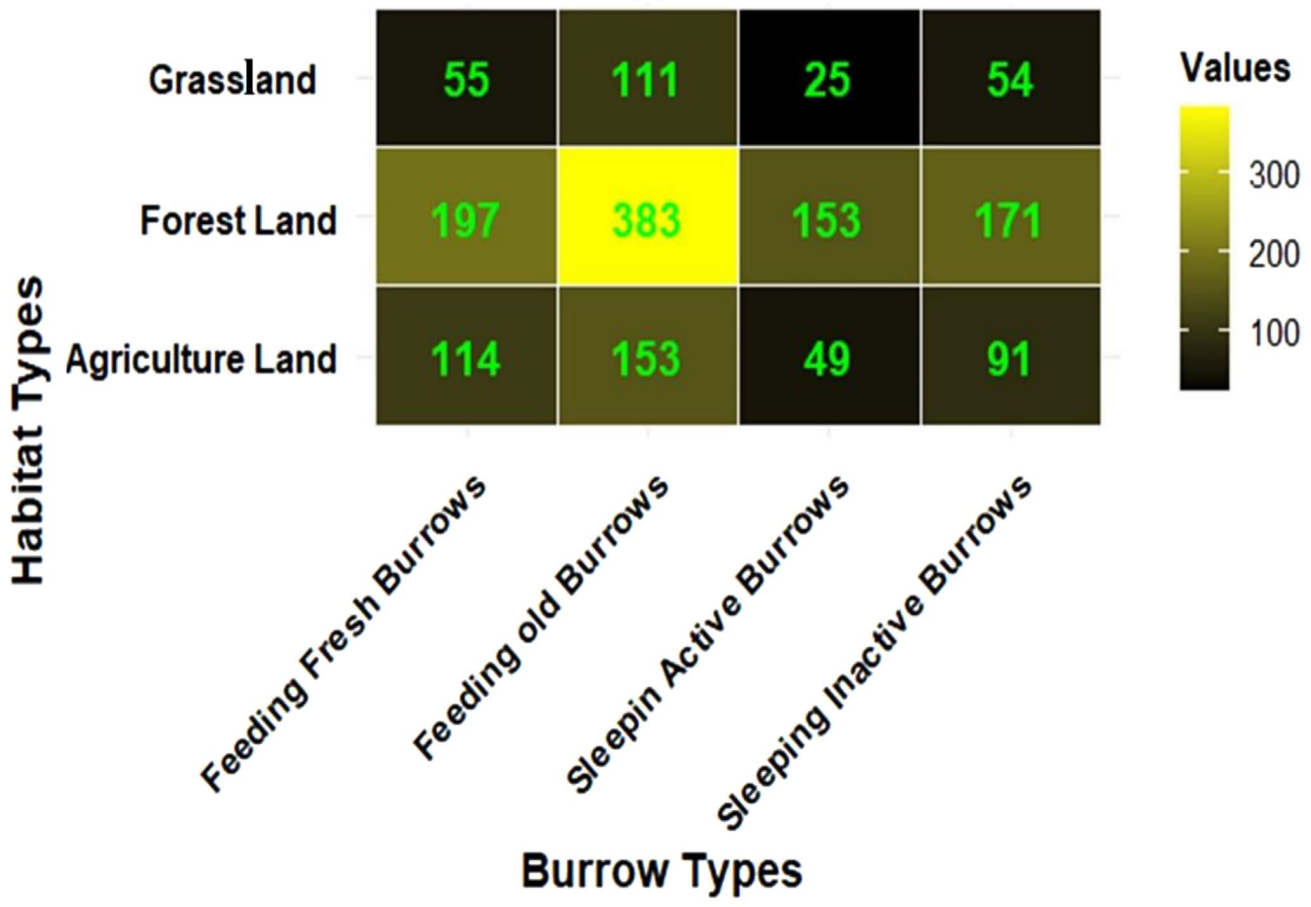
Heatmap of burrow distribution across habitat Types, offering insights into environmental preferences and interactions of Indian pangolins. “Values” represent the number of burrows recorded in each habitat–burrow category.

Four distinct burrow types were identified during field surveys: feeding fresh, feeding old, sleeping active, and sleeping inactive. During our survey, active burrows were recognized by fresh signs of use, including clear footprints, claw marks, feces, and the presence of termites or ant bodies at the entrance. Inactive burrows lacked such signs and were often covered by spider webs, grasses, and tree leaves found around the entrance. Feeding fresh burrows were characterized by deep, newly excavated, circular holes with signs of fresh foraging activity. Feeding old burrows exposed evidence of past foraging, but they were weathered, with minimal recent disturbance. Feeding burrows, in contrast, have smaller entrances and significantly less depth compared to resting burrows. During the survey, 102 sites across six districts recorded 227 active burrows and 316 inactive burrows. We also observed 366 fresh‐feeding burrows and 647 old‐feeding burrows. This data provides a comprehensive view of burrow activity across the studied areas. Chi‐squared tests revealed significant changes in burrow distribution across habitat categories (*χ* ^2^ = 17.756, df = 6, *p* < 0.01). These results indicate that different habitat types host varying quantities of active, inactive, fresh‐feeding, and old‐feeding burrows. A heatmap was created to visually display these distribution patterns, highlighting different habitat types with strong pangolin activity (Figure 5).

### 3.2 Vegetation Preferences and Habitat Characteristics of the Indian Pangolin

**FIGURE 6:**
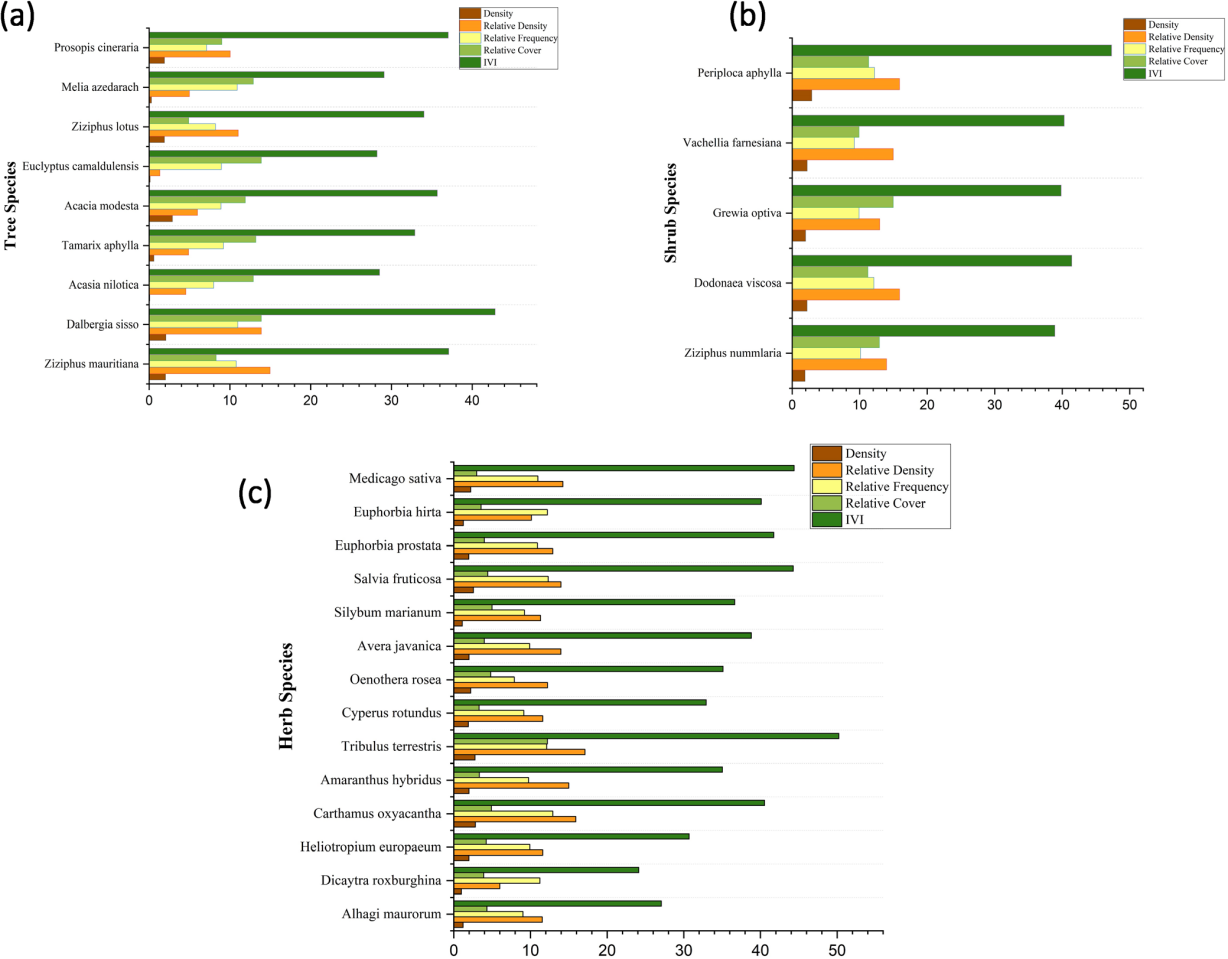
Density, relative density, relative frequency, relative cover, and importance value index (IVI) of vegetation in the habitat of the Indian pangolin (*Manis crassicaudata*): (A) tree species, (B) shrub species, and (C) herb species at selected sites.

**FIGURE 7:**
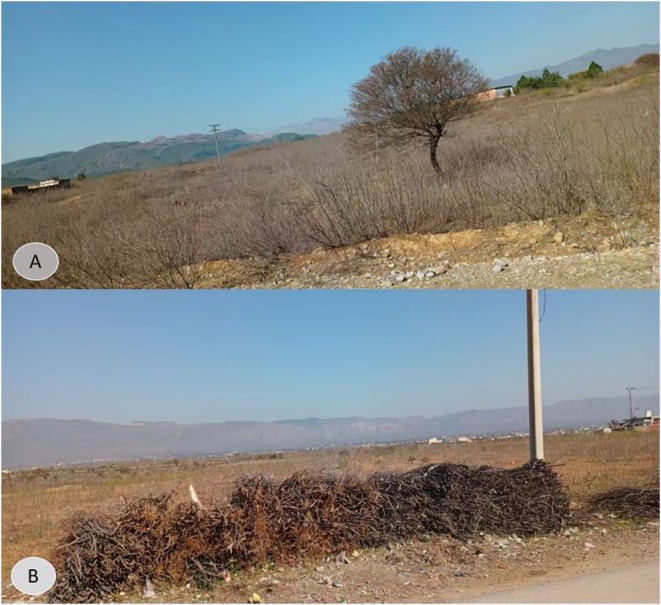
(A) shows the natural habitat with *Acacia nilotica*, which provides shelter and resources for the Indian Pangolin (*Manis crassicaudata*). (B) shows the removal of this plant by local communities, which reduces habitat quality for the pangolins.

Detailed vegetation analysis revealed pangolins' great preference for certain tree species, particularly *Dalbergia sisso*, which had the highest importance value index (IVI = 42.86) (Figure 6). In contrast, *Eucalyptus camaldulensis* had the lowest preference (IVI = 28.323), which could be attributed to less favorable environmental conditions. The average tree density was 1.32 ± 0.34, with a total relative frequency of 9.22 ± 0.46. Shrubs had a mean density of 2.24 ± 0.17 and a total relative frequency of 10.70 ± 0.61, whereas herbs had a mean density of 1.91 ± 0.15 and a total relative frequency of 10.53 ± 0.40. The examination of tree, shrub, and herb layers revealed varying density and frequency, with specific plant types, such as *Acacia nilotica* , receiving higher preference values, indicating their importance in providing shelter and protection from predators (Figure 7).

### 3.3 Habitat Suitability Modeling With MaxEnt

#### 3.3.1 Model Performance and Environmental Variables

**FIGURE 8:**
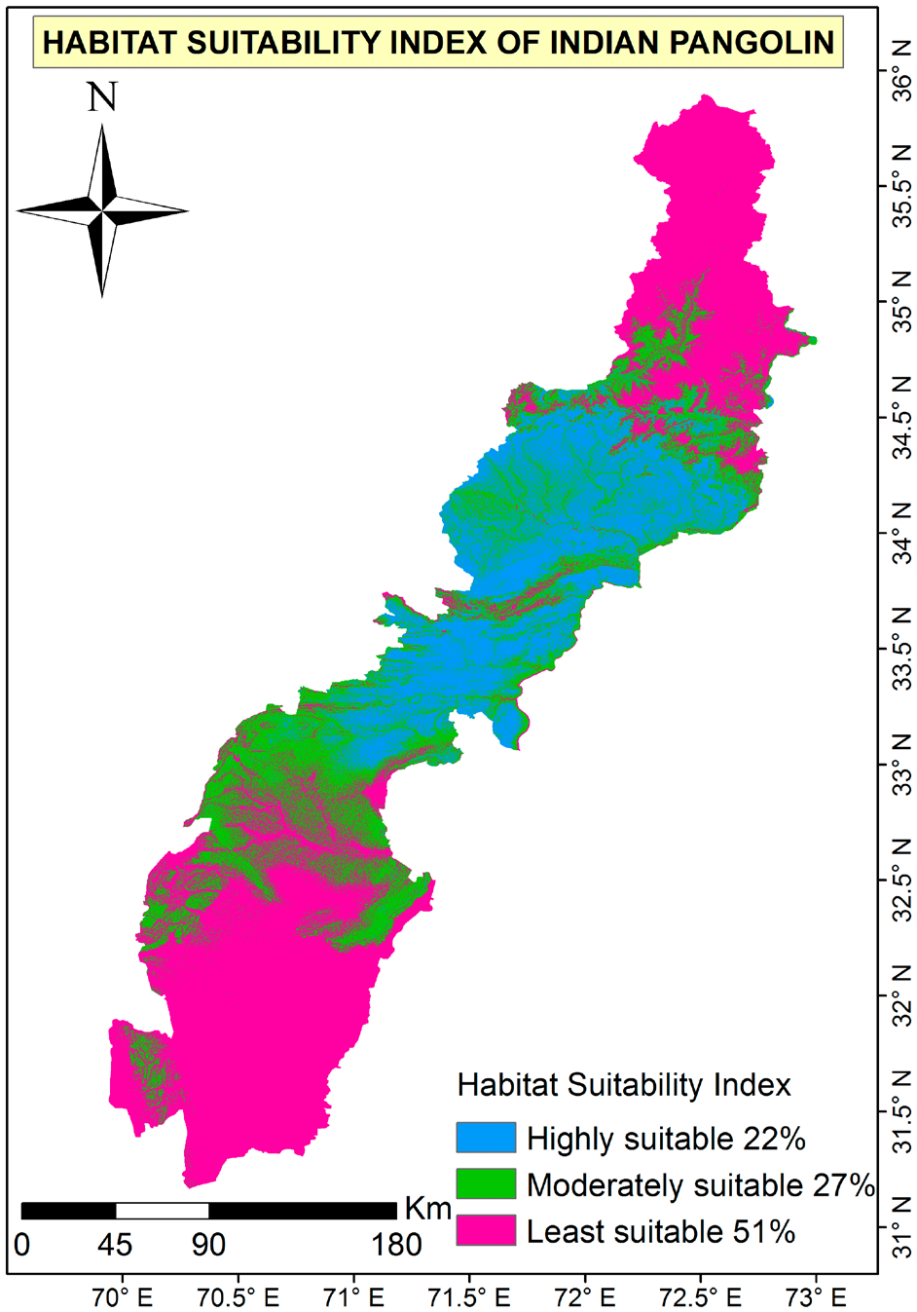
The habitat suitability map illustrates the spatial distribution of suitable habitats for the Indian pangolin in Western Pakistan. Areas with high suitability are depicted in blue, moderate suitability in green, and least suitability in red.

**FIGURE 9:**
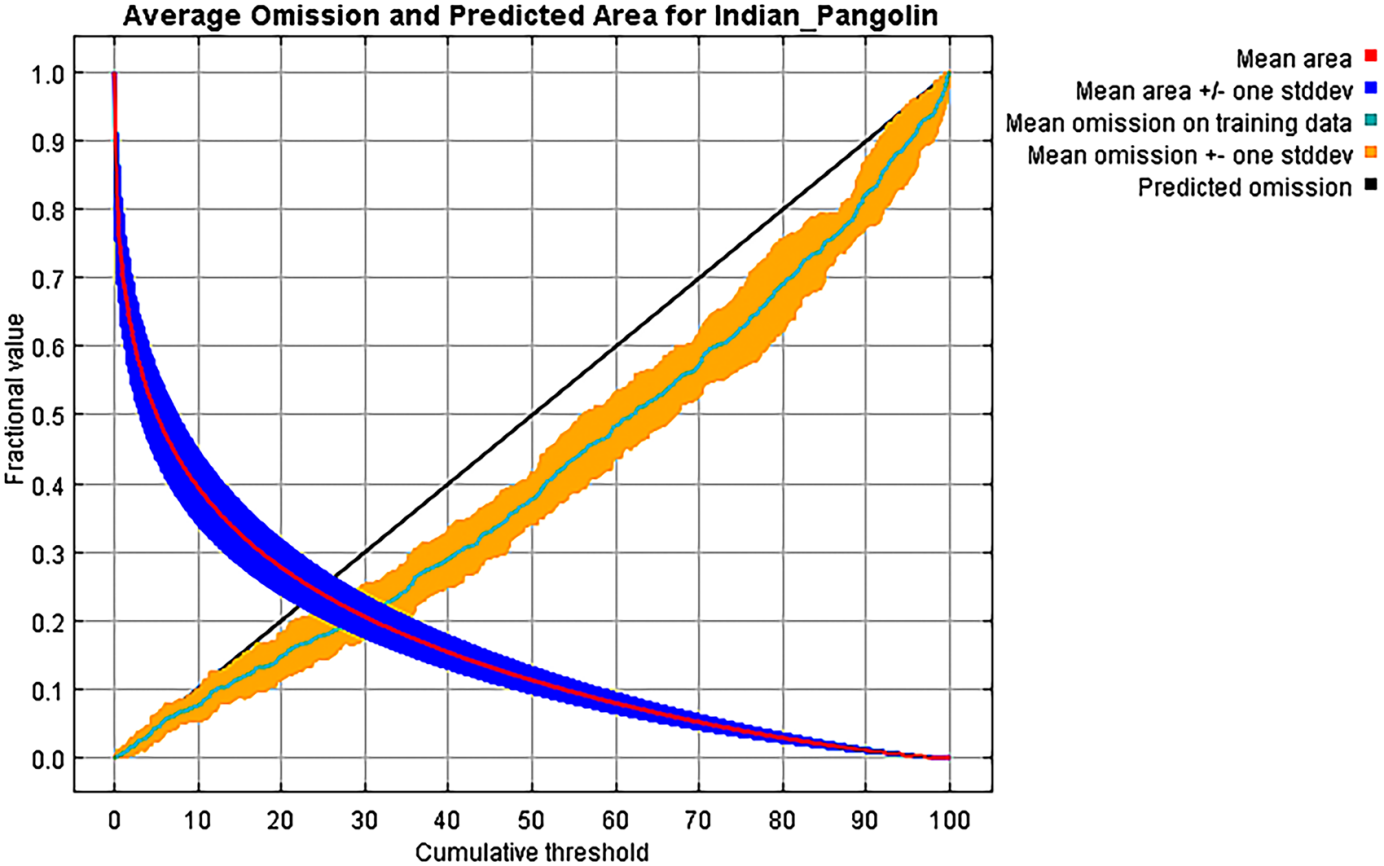
Threshold‐dependent validation of MaxEnt model for Indian pangolin habitat suitability.

**FIGURE 10:**
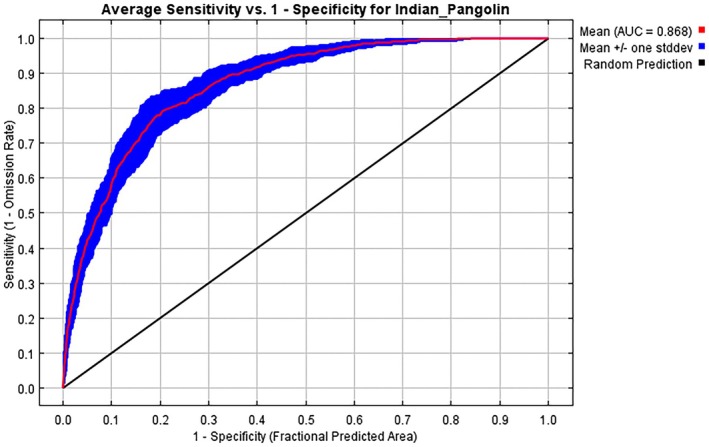
ROC curve and AUC values of the MaxEnt Model predict the accuracy of the habitat suitability model, affirming the utility of the environmental variables selected.

The MaxEnt model generated a precise habitat suitability map for Indian pangolins in the KP province. Based on 15 bootstrap repetitions, the model achieved good prediction accuracy, with an average AUC of 0.868 and a standard deviation of 0.020 (Figures 8, 9, 10). Model accuracy was very good in terms of TSS (0.674). The model's performance for the Indian pangolin is good, with an AUC_Diff_ value of 0.050, indicating a well‐fitted and reliable model. The analysis found that 22% (1674 km^2^) of the overall study area was classified as highly suitable habitat, 27% (3851.7 km^2^) as moderately suitable habitat, and 51% (8438.3 km^2^) as least suitable habitat.

**FIGURE 11:**
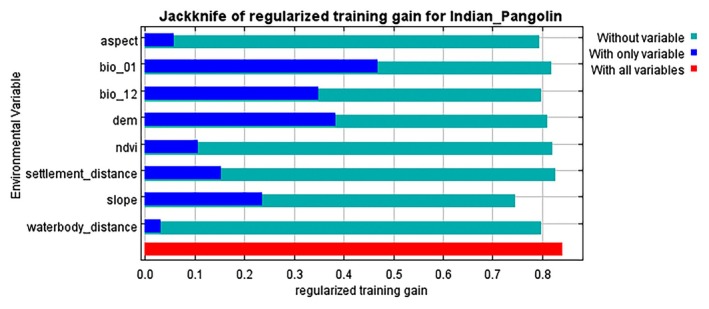
Jackknife analysis of environmental variables impacting indian pangolin habitat suitability.

The jackknife test for variable relevance revealed that Bio_12 (annual precipitation) was the most significant predictor variable, accounting for 35.7% of the model's predictive power. Slope and DEM also had important roles, with 23.8% and 18.6%, respectively (Table 2). The jackknife test of variable importance showed that the environmental variable with the highest gain when used in isolation is bio_01, which therefore appears to have the most useful information by itself. The environmental variable that decreases the gain the most when it is omitted is slope, which therefore appears to have the most information that isn't present in the other variables (Figure 11). Table 2 summarizes the contribution and permutation importance of each environmental variable.

**TABLE 2:** Relative contributions and permutation importance of environmental variables.

Variable	Percent contribution	Permutation importance (%)
Bio_12 (precipitation)	35.7	24.1
Slope	23.8	20.8
DEM	18.6	23.2
Bio_01 (temperature)	7.9	13.7
Aspect	5.8	4.8
Waterbody distance	4.3	6.6
NDVI	2.1	4.9
Settlement distance	1.8	1.9

**FIGURE 12:**
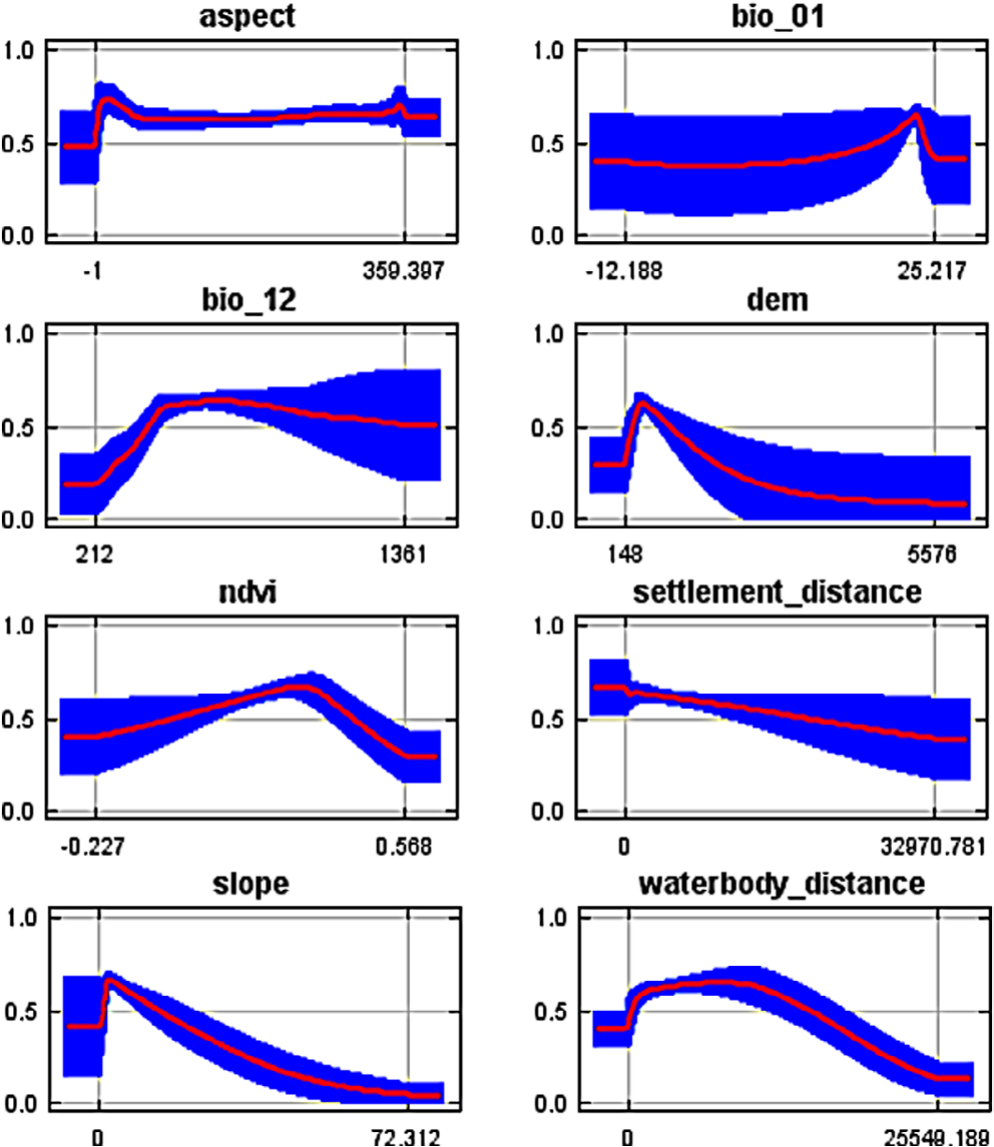
Response curves for key environmental variables.

Response curves for each environmental variable were created to demonstrate their impact on habitat suitability. Response curves indicated that habitat suitability for the Indian pangolin increases abruptly as annual precipitation rises from 200 mm to 430 mm, followed by a slight increase up to 700 mm, and then gradually decreases as precipitation reaches 1400 mm, peaking at a suitability value of 0.65 at 700 mm. Habitat suitability decreases with increasing slope, peaking at a value of 0.68 at gentle slopes (0°–5°). Similarly, habitat suitability peaks at an elevation of 500 m with a value of 0.62 and declines with further elevation increase. These findings indicate that moderate precipitation, gentle slopes, and the positive influence of pangolin presence (Figure 12).

### 3.4 Comparative Performance of Algorithms

**FIGURE 13:**
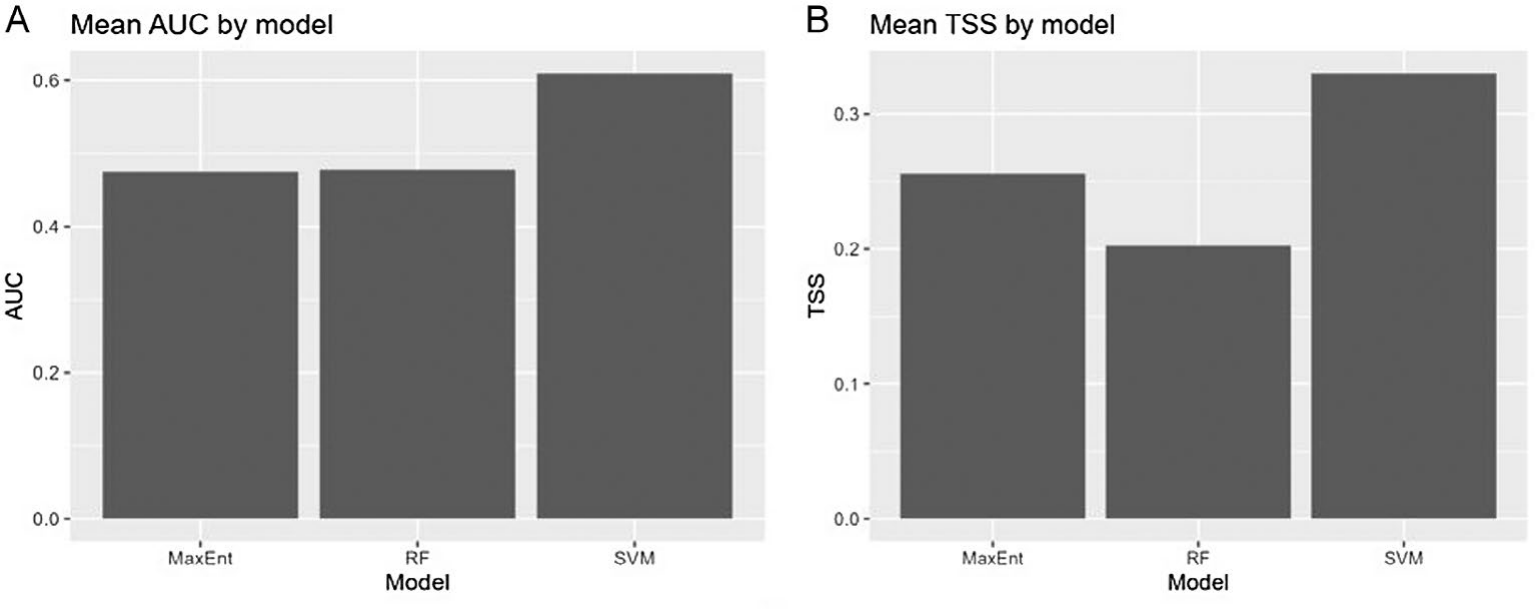
Comparative model performance. (A) Mean AUC by model; (B) mean TSS by model. Bars show means across spatial folds (out‐of‐fold).

Across the spatial fivefold evaluation, SVM achieved the highest discrimination (mean AUC ≈ 0.61; mean TSS ≈ 0.33), whereas RF and MaxEnt performed slightly lower (AUC ≈ 0.48–0.50; TSS ≈ 0.20–0.25; Figure 13; Table 3). Precision–recall performance followed the same ranking (Table 3). Out‐of‐fold model probabilities combined into a TSS‐weighted ensemble yielded a small stability gain relative to individual models (Table S1). Variable‐importance analyses indicate that Elevation_m, NDMI, and NDWI were consistently influential across learners (RF impurity importance; SVM and MaxEnt permutation importance; Table S2). Partial‐dependence curves reveal clear non‐linear responses, notably mid‐elevation optima and hump‐shaped relationships for NDMI/NDWI (Figures S1–S3). Elevation was the strongest driver of AUC, with moisture indices (NDWI/NDMI) and mean temperature adding moderate gains, while NDVI, precipitation, and diurnal range had smaller effects (Table S3). The reliability curve (Figure S4) shows moderate deviation from the 1:1 line, representing partial miscalibration between predicted suitability and observed occurrences.

**TABLE 3:** Out‐of‐fold (OOF) model performance comparison showing mean AUC, TSS, and PR‐AUC across five folds for MaxEnt, Random Forest (RF), Support Vector Machine (SVM), and the ensemble model.

Model	AUC	TSS	PRAUC
MaxEnt	0.474619	0.255764	0.280352
RF	0.477222	0.202301	0.248426
SVM	0.609797	0.330314	0.282993
Ensemble (OOF)	0.290338	0.012703	0.192433

*Note:* SVM achieved the highest discrimination performance, followed by MaxEnt and RF.

### 3.5 Public Perception, Hunter, Provincial Layers, and Illegal Trade Routes

Our results show that local knowledge about Indian pangolin trafficking was widespread across the 15 surveyed districts. Out of 255 questionnaires and 196 interviews, approximately 72% of respondents confirmed awareness of illegal pangolin trade activities. Many provided detailed information about commonly used trade routes and techniques for transporting both small and live animals. The maps identified urban centers such as Rawalpindi, Lahore, Peshawar, and Quetta as central hubs in the illegal trade network. These markets showed consistent trafficking activity, which helps identify critical locations for targeted conservation efforts.

### 3.6 Habitat Threats to the Indian Pangolin

**FIGURE 14:**
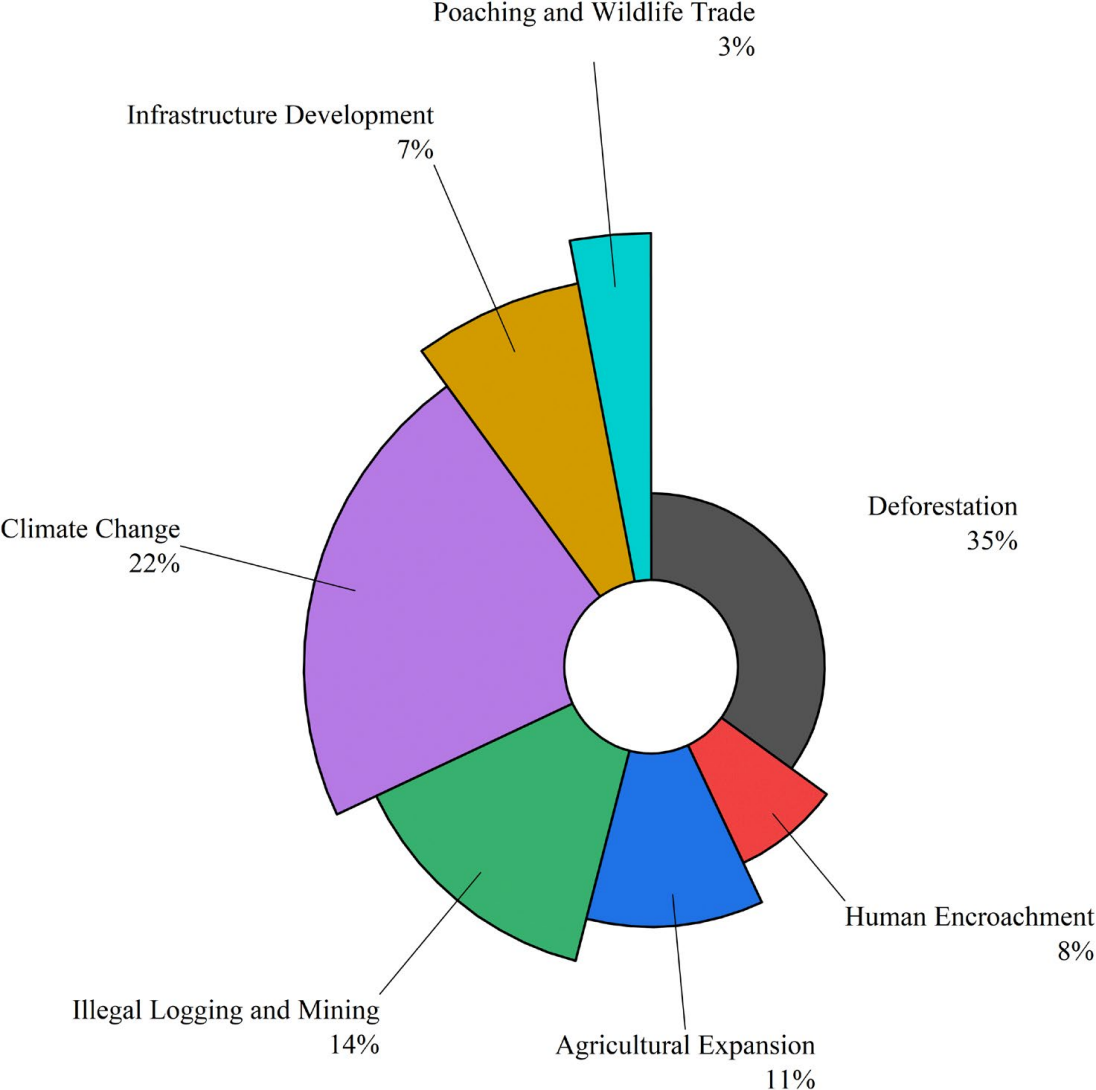
Comparative analysis of perceived threats for Indian pangolins based on community responses. “Threats” include habitat degradation and illegal activities.

**FIGURE 15:**
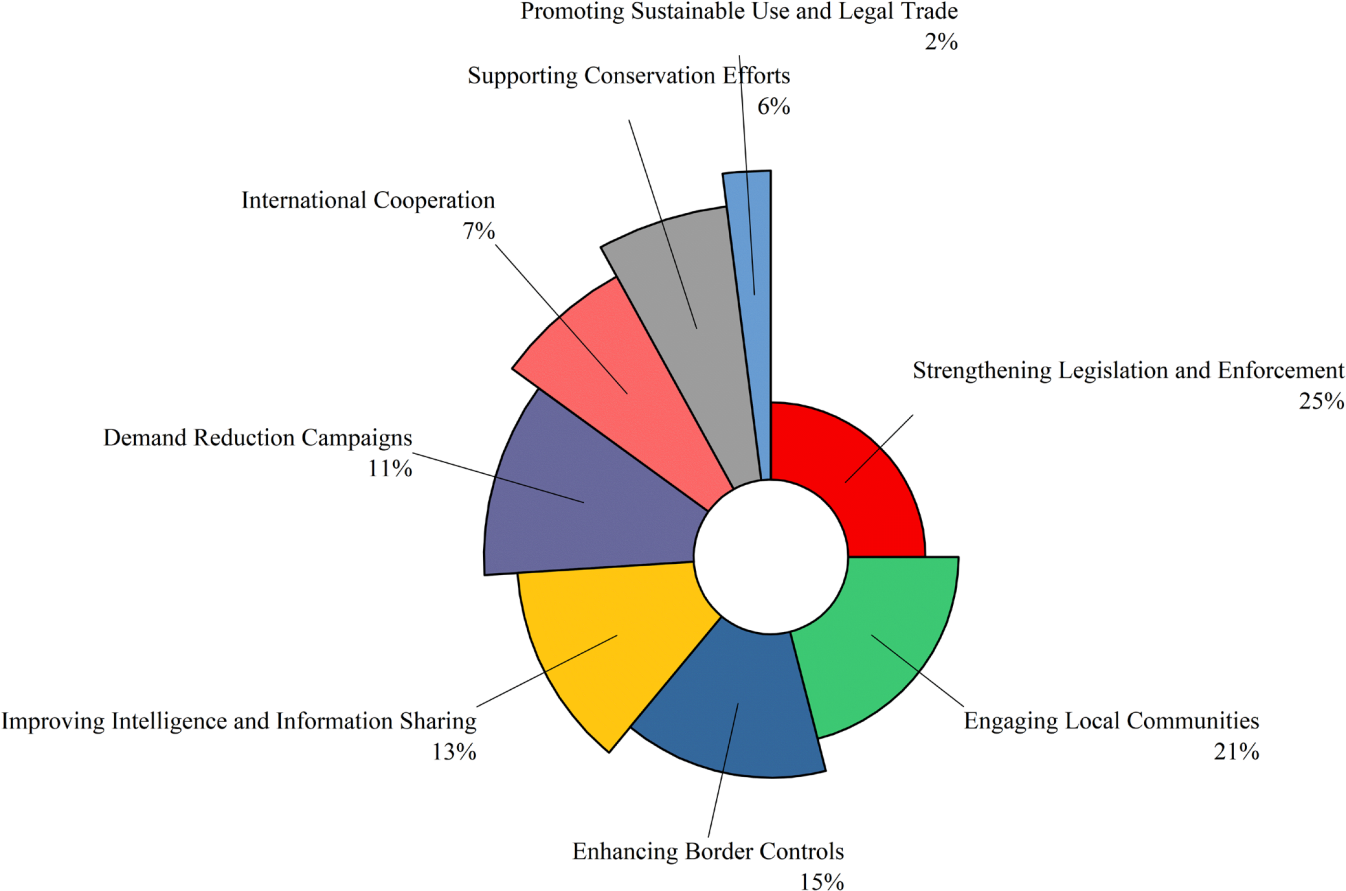
Comparative analysis of perceived conservation measures for Indian pangolins based on community responses.

**FIGURE 16:**
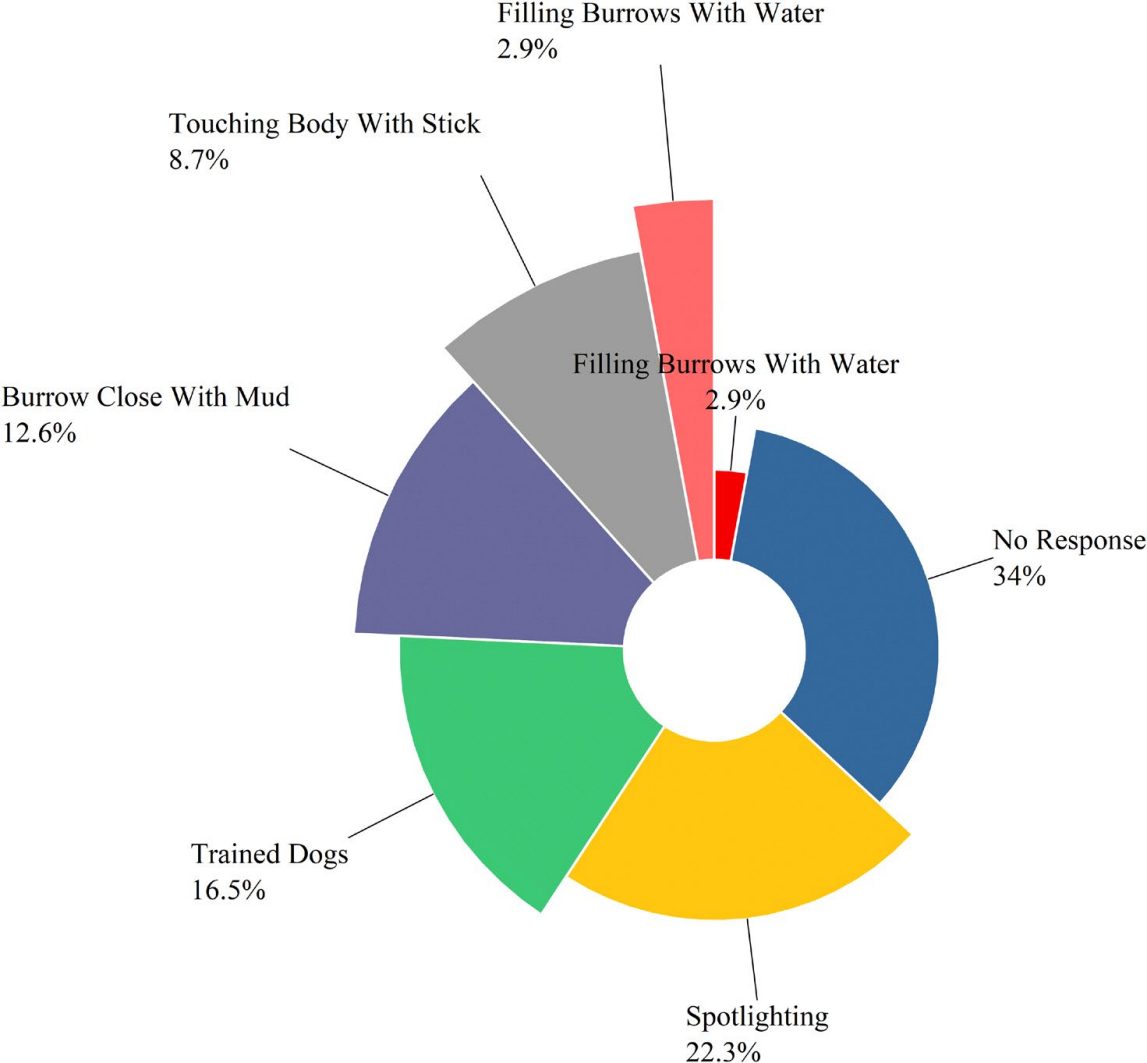
The capturing techniques of Indian pangolins in selected sites.

Respondents identified deforestation (35%), climate change (22%) as the major threats to the Indian pangolin, while poaching only contributed 3% of respondents, likely reflecting limited public awareness rather than a minor role of poaching in pangolin decline (Figure 14). Key conservation measures included strengthening legislation and enforcement (25%), engaging local communities (21%), and enhancing border controls (15%) (Figure 15). Lesser‐emphasized categories involved climate change, infrastructure development, and international cooperation. The findings highlight the need for integrated conservation strategies focused on habitat protection and law enforcement. In contrast, “deforestation” was recognized as the most severe habitat concern, accounting for 35% of all factors affecting pangolin habitats. The graph shows the relative frequency of community responses regarding conservation actions and perceived environmental threats to Indian pangolins. A total of 71 pangolins were reported to be killed by members of the local communities where the respondents reside. Approximately 35% did not specify their interactions, while spotlighting (23%), trained dogs (17%), and other methods, such as touching pangolins with sticks (9%) and filling burrows with water (3%) were reported (Figure 16).

## 4 Discussion

In Pakistan, the Indian pangolin is protected under the Khyber Pakhtunkhwa Province Wildlife (Protection, Preservation, Conservation, and Management) Act, 1975, and the Islamabad Wildlife (Protection, Preservation, Conservation, and Management) Ordinance, 1979. It is included in the third schedule of the Punjab Wildlife Act, 1974 (Amendment 2007). According to the third schedule, it is protected throughout the year. The species has also been included in Appendix I of the CITES since 2016, which prohibits any worldwide trade of the species and its products (scales) legally. As a CITES participant, Pakistan aligns its national legislation with these international regulations. This supports the species' protection under both local and global conservation laws. A study by Waseem et al. (2020) found evidence of pangolin occurrence in three different types of habitats in their study area, that is, agricultural land, natural forests, and grassland; however, their results indicated that the natural forest is the preferred habitat of the pangolin. This preference indicates that, compared to grassland and agricultural land, more cover might be available to the Indian pangolin. The occurrence of pangolin in several types of tropical forests, as well as grasslands, open land, and degraded habitats, including near villages (Yuvraj 2024). Indian pangolin can also adjust to modified habitats depending upon certain factors, in subtropical thorn forests and desolate undulating areas (Roberts 1997). Similarly, Pai (2008) also confirmed tropical forests in the habitats of the species, ranging from dry deciduous to moist, thorn‐plus grassland.

Our findings on pangolin preference for specific tree species, such as *Dalbergia sisso* for shelter, are consistent with the findings of Khattak et al. (2023), who observed similar preferences in pangolins in Southeast Asian forests due to the tree's dense foliage and branch structure, which provides optimal protection and foraging opportunities. Similar to recent research conducted in Nepal (Rai et al. 2019; Suwal et al. 2020), which revealed that pangolins prefer habitats with medium canopy coverage over forests that are too dense or too sparse, burrows were usually detected in areas with canopy cover between 51% and 75%. Mahmood et al. (2021) confirm that the Indian Pangolin favors natural wild regions (55.88%) over human vicinity areas (24%) and agricultural land (20.11%). In our study, similar to the findings by Waseem et al. (2020), although pangolins showed a clear preference for forest habitats (61.3%), the notable presence of signs in agricultural lands (32.11%) shows that these modified areas also provide important habitats and should be considered in conservation planning and management strategies. Natural forests must be protected because Indian pangolins mostly prefer natural forests. Khattak et al. (2023) conducted a study on Margalla Hills National Park and reported that these declines are caused not just by direct human acts but also by indirect factors such as land use changes and agricultural intensification, both of which impair the pangolin's native habitat. Ravine habitats in the Himalayan foothills, as well as places like the Margalla Hills National Park, have been especially heavily damaged.

Our data show that burrow forms vary significantly across different habitat types, which is consistent with global studies on habitat specificity in pangolin species. The higher pangolin activity observed in central regions may be influenced by sampling distribution and data availability, which has been acknowledged as a study limitation. The suitable habitat is still available for Indian pangolin in the Potohar Plateau, and in the AJ&K part of Pakistan, they also collected data about active and inactive (old) burrows of the species in the study area (Waseem et al. 2020). According to Waseem et al. (2020), a large number of pangolin burrows (both feeding burrows and sleeping) were found inactive; however, there were also active burrows present in the study area. This specifies the presence of the species in the AJ&K and Potohar Plateau, Pakistan. Analysis of the presence/absence data indicates that approximately 32% of the study area is occupied by Indian pangolins (Waseem et al. 2020). Indian pangolin occupied a total of 89% of the area of the Potohar Plateau (19,854 km^2^) in 2011–2013, whereas IUCN data reported 71% (15,801 km^2^) area of the Potohar Plateau occupied previously by Indian pangolin (Mahmood et al. 2019). Four bioclimatic variables, including the mean temperature of the coldest quarter (bio12), annual mean temperature (bio1), precipitation seasonality (bio15), and temperature seasonality (bio4), are the most important in describing Indian pangolin occurrence (Qasim et al. 2024). However, annual mean temperature (bio1) had the greatest contribution in describing the distribution of the Indian pangolin, followed by the mean temperature of the coldest quarter; temperature seasonality, and precipitation seasonality contributed the least to the maxent model. Looking into these prior records of the existence of the Indian pangolin, the current study reports a likely shrinkage of the suitable habitat of the species in the studied region. Our Maxent approaches reveal that 22% of the habitat is classified as highly suitable, 27% as moderately suitable, and the remaining 51% as marginally suitable. These findings highlight varying degrees of habitat suitability across the landscape, providing critical insights for conservation and 23 management strategies.

The use of MaxEnt modeling in this work demonstrates the predictive potential of environmental variables on habitat suitability for Indian pangolins. Our findings on the importance of yearly precipitation and vegetation density are supported by Phillips et al. (2019), who found comparable effects in their ecological niche modeling of pangolins in tropical settings. We conducted our research work in 15 districts of the Khyber Pakhtunkhwa (KP) province of Pakistan. Our study's emphasis on slope and elevation is consistent with the findings of Shilereyo et al. (2021), who found these parameters crucial in determining habitat suitability for wildlife in mountainous environments.

Our analysis of illegal trade routes and the prevalence of poaching activities provides critical insights that are consistent with broader trends observed by Challender and Hywood (2011), in which illegal wildlife trade continues to decimate pangolin populations despite international conservation initiatives. Waseem et al. (2020) conducted a survey (*N* = 239) of shops, including animal and pet shops, birds, and local Hakeem, Pansari (herbal medicine vendors), and street vendors from five metropolitan cities, viz. Rawalpindi, Karachi, Peshawar, Lahore, and Muzaffarabad exposing their involvement in the illegal trade of wildlife species, even though some markets catered to the legal pet trade in bird species. We identified a few merchants in Thokar Niaz Baig, Lahore city, who are involved in large‐scale trafficking of pangolin meat and scales within the city and outside the city as well. These dealers sell the pangolin scales at varying prices, ranging between Rs. 17,000 and 18,000/kg to Chinese nationals visiting Pakistan and residing in the Defense Housing Authority (DHA) Lahore. The scales are usually used in traditional medicine, are supposed to possess healing properties and are also used in certain cultural practices. Our findings demonstrate the need for improved legal frameworks and tighter enforcement, as noted by Challender et al. (2015), emphasizing the importance of incorporating these measures with community‐based conservation methods. These findings underscore the diverse practices within communities and emphasize the need for targeted conservation education and interventions to mitigate threats to pangolins.

Ahmad and Li (2024) reported that 179 Indian pangolins were killed in Khyber Pakhtunkhwa, while in Punjab, only 59 were killed (Attock, Chakwal, Jhelum, and Rawalpindi). Many studies have described the pangolin trade, particularly from Attock, Chakwal, Jhelum, and Rawalpindi (Mahmood et al. 2019). From January 2011 to May 2012 in the Pothwar Plateau, 118 Indian pangolins were caught and illegally killed (Mahmood et al. 2012). This study adds a crucial dimension to our understanding of pangolin conservation in Khyber Pakhtunkhwa and contributes to the regional conservation data set. The techniques and conclusions of this study can inform targeted conservation measures, as proposed by Mahmood et al. (2016), who stress specific habitat protection and restoration strategies to reduce concerns about human encroachment and agricultural growth.

To effectively maintain Indian pangolin habitats in Khyber Pakhtunkhwa, a comprehensive approach that includes local community engagement, stronger law enforcement, and increased scientific study is required. Initiatives should prioritize alternative livelihoods, improve wildlife law enforcement skills, and implement enhanced surveillance to minimize poaching. More scientific research is needed to better understand pangolin ecology and behavior, which will guide the establishment and maintenance of protected areas and conservation corridors. It will be critical to integrate habitat conservation into regional and national policy, strengthen cross‐border collaboration, and develop environmental education programs. These initiatives aim to create a long‐term framework for conserving Indian pangolins while also aligning with global biodiversity conservation aims.

Our comparative analysis demonstrates that key suitability patterns are not an artifact of MaxEnt alone: independent algorithms (RF, SVM) trained with identical predictors and spatial folds recover similar signals and non‐linear responses, corroborated by a simple TSS‐weighted ensemble. While the current predictors include concise bioclimatic proxies rather than full gridded layers, the agreement across algorithms strengthens confidence in the overall habitat signal and provides interpretable, non‐linear response curves for the most influential predictors (Elevation_m, NDMI, NDWI). Once final bioclimatic layers are available, the same framework can be re‐run to refine estimates without altering the study design. A limitation of this study is that it did not account for spatial autocorrelation in environmental variables (e.g., slope, NDVI, distance metrics). Although Pearson's correlation analysis helped minimize overfitting and ensured model robustness, and multicollinearity was addressed for climatic predictors, Moran's I was not applied to assess spatial autocorrelation, which should be addressed in future analyses.

## 5 Conclusion

This study provides a detailed assessment of Indian pangolin habitat suitability and associated conservation challenges in Khyber Pakhtunkhwa. Distribution modeling revealed fragmented habitat availability, with only 22% of the region classified as highly suitable. Significant variation in burrow distribution across habitats indicates specific ecological preferences. Although community awareness of illegal trade appeared limited, field evidence and qualitative insights confirmed its ongoing presence. Respondents highlighted the need for stronger legislation and enforcement. These findings underscore the urgency of integrating spatial planning, enforcement, and community‐based conservation to ensure effective protection of the species.

## Author Contributions

**Tariq Ahmad:** formal analysis (equal), investigation (equal), methodology (equal), resources (equal), software (equal), writing – original draft (equal), writing – review and editing (equal). **Arshad Ali:** formal analysis (equal), investigation (equal), software (equal). **Muhammad Farooq:** data curation (equal), investigation (equal), methodology (equal). **Bo Li:** conceptualization (equal), methodology (equal), software (equal), supervision (equal), writing – review and editing (equal). **Sayantani M. Basak:** data curation (equal), validation (equal), writing – review and editing (equal). **Tika Ram Poudel:** formal analysis (equal), funding acquisition (equal), project administration (equal). **Khuzin Dinislam:** conceptualization (equal), formal analysis (equal), software (equal).

## Funding

The study was supported by the National Basic Resources Investigation Program (2023FY100405).

## Ethics Statement

This study involved non‐invasive field observations and questionnaire‐based interviews with local community members. Under our institution's policies, formal ethical approval was not required for these activities.

## Consent

Informed consent was obtained from all participants, and participation was voluntary and anonymous.

## Conflicts of Interest

The authors declare no conflicts of interest.

## Supporting information


**Data S1:** ece372610‐sup‐0001‐DataS1.docx.

## Data Availability

All data, metadata, and relevant code supporting the findings of this study are provided as Supporting Information and are available with this submission.
