# Supplementary material for: Exploring Habitat Preferences, Suitability, and Illegal Trade Routes of Indian Pangolins in Western Pakistan: Implications for Conservation
Source: Ecol Evol. 2026 Jan 9;16(1):e72610. doi: 10.1002/ece3.72610 (PMC12788983; doi:10.1002/ece3.72610)
Supplement: Supplementary file 1 — Data S1: ece372610‐sup‐0001‐DataS1.docx. [file ECE3-16-e72610-s001.docx]

**Table S1.** TSS-weighted ensemble performance (OOF) with AUC, TSS, and PR-AUC for the ensemble and individual models.

| Model | AUC | TSS | PRAUC |
| --- | --- | --- | --- |
| MaxEnt | 0.474619 | 0.255764 | 0.280352 |
| RF | 0.477222 | 0.202301 | 0.248426 |
| SVM | 0.609797 | 0.330314 | 0.282993 |
| Ensemble (OOF) | 0.290338 | 0.012703 | 0.192433 |

**Table S2.** *Variable importance across algorithms.*
RF impurity importance; SVM and MaxEnt permutation AUC-drop.

| **variable** | **perm_AUC_drop** |
| --- | --- |
| NDVI | 0.024459459 |
| Elevation_m | 0.010506757 |
| NDWI | 0.009684685 |
| BIO1_mean_temp_C | 0.006092342 |
| NDMI | 9.23E-04 |
| BIO2_diurnal_range_C | -4.73E-04 |
| Precipitation | -9.57E-04 |

**Table S3.** Pairwise DeLong tests (AUC) between models.

| **variable** | **impurity** |
| --- | --- |
| Elevation_m | 24.37632462 |
| NDWI | 19.59612465 |
| NDMI | 19.38419555 |
| BIO1_mean_temp_C | 18.47760823 |
| NDVI | 16.76401152 |
| Precipitation | 16.70339205 |
| BIO2_diurnal_range_C | 15.99816914 |


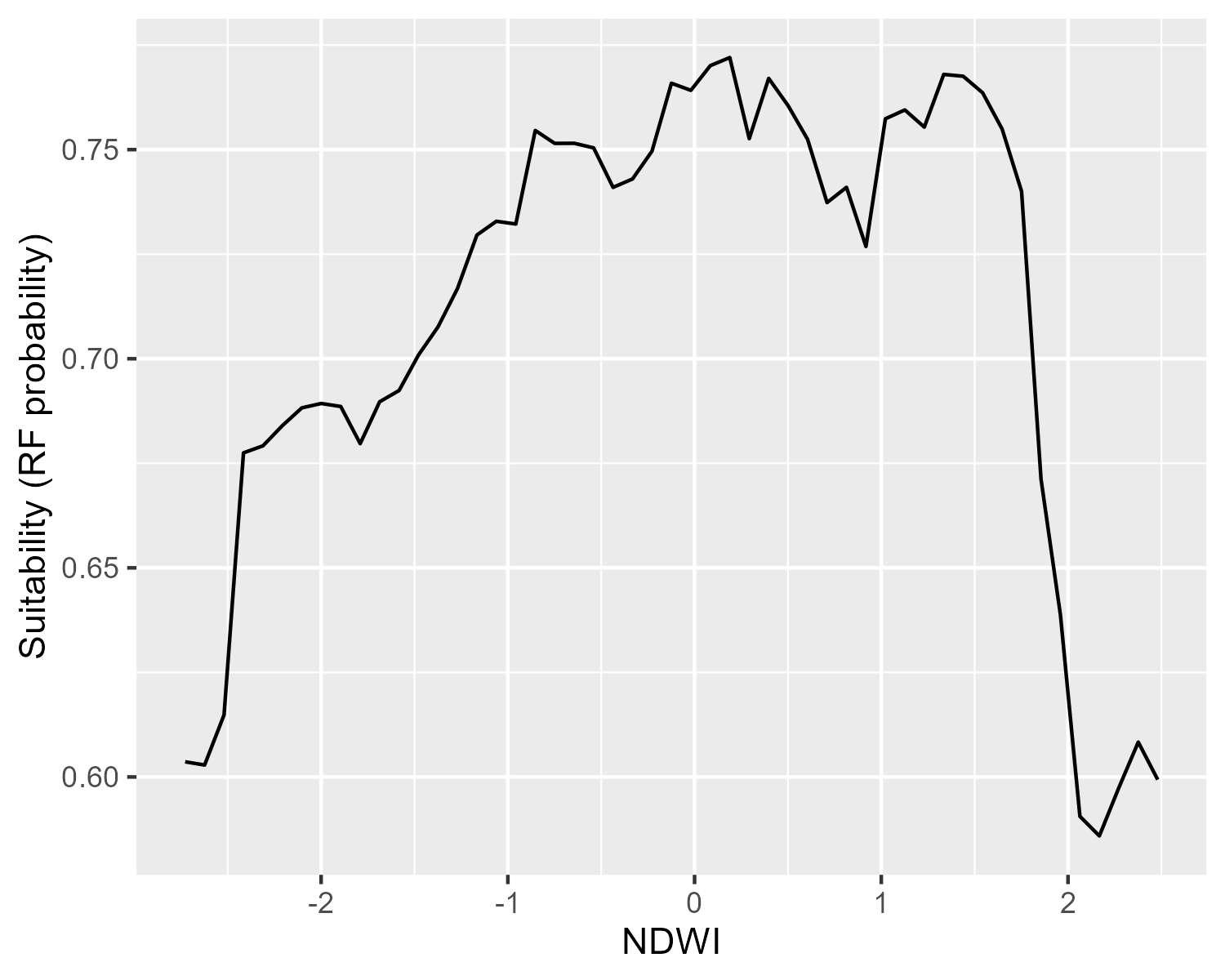

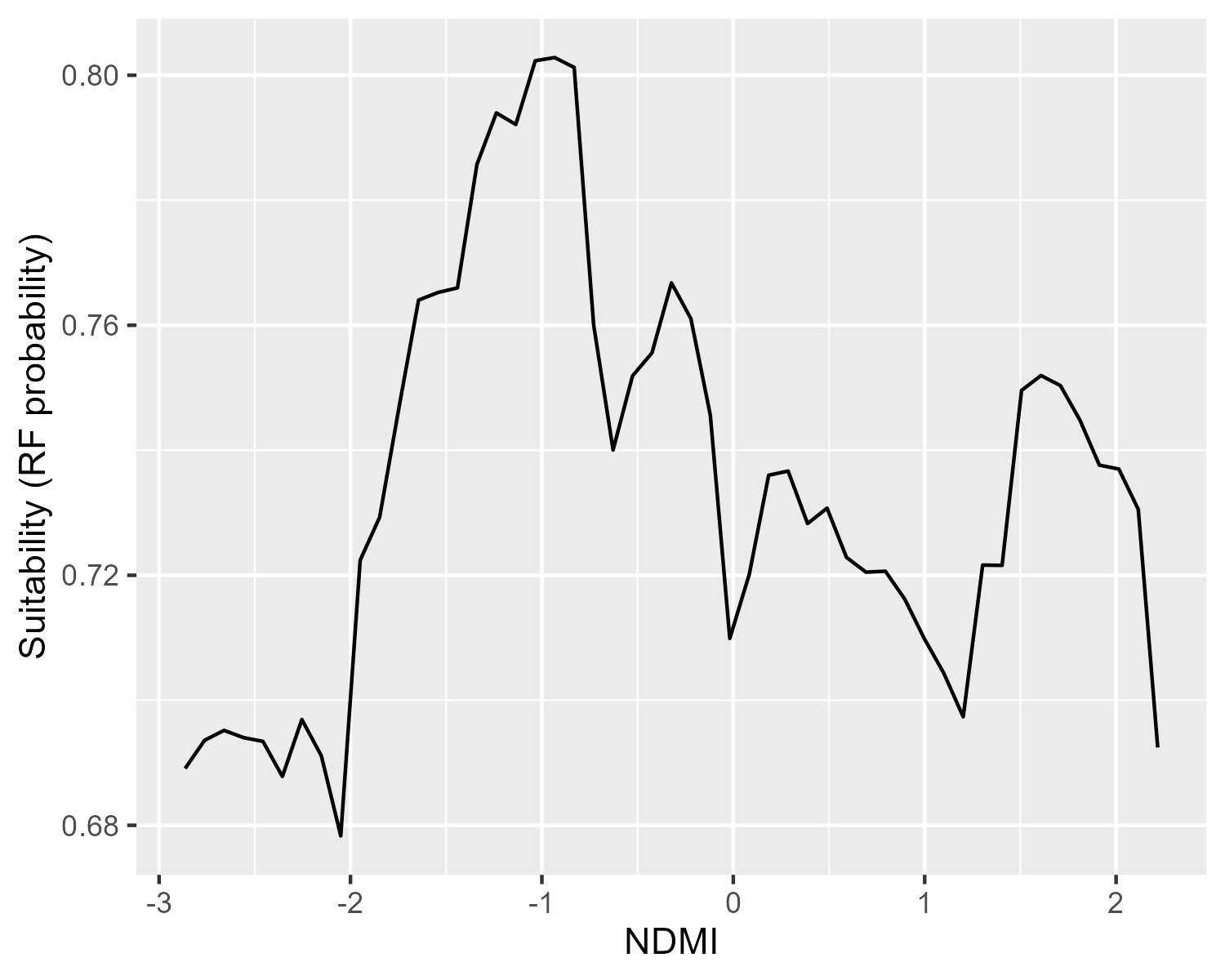

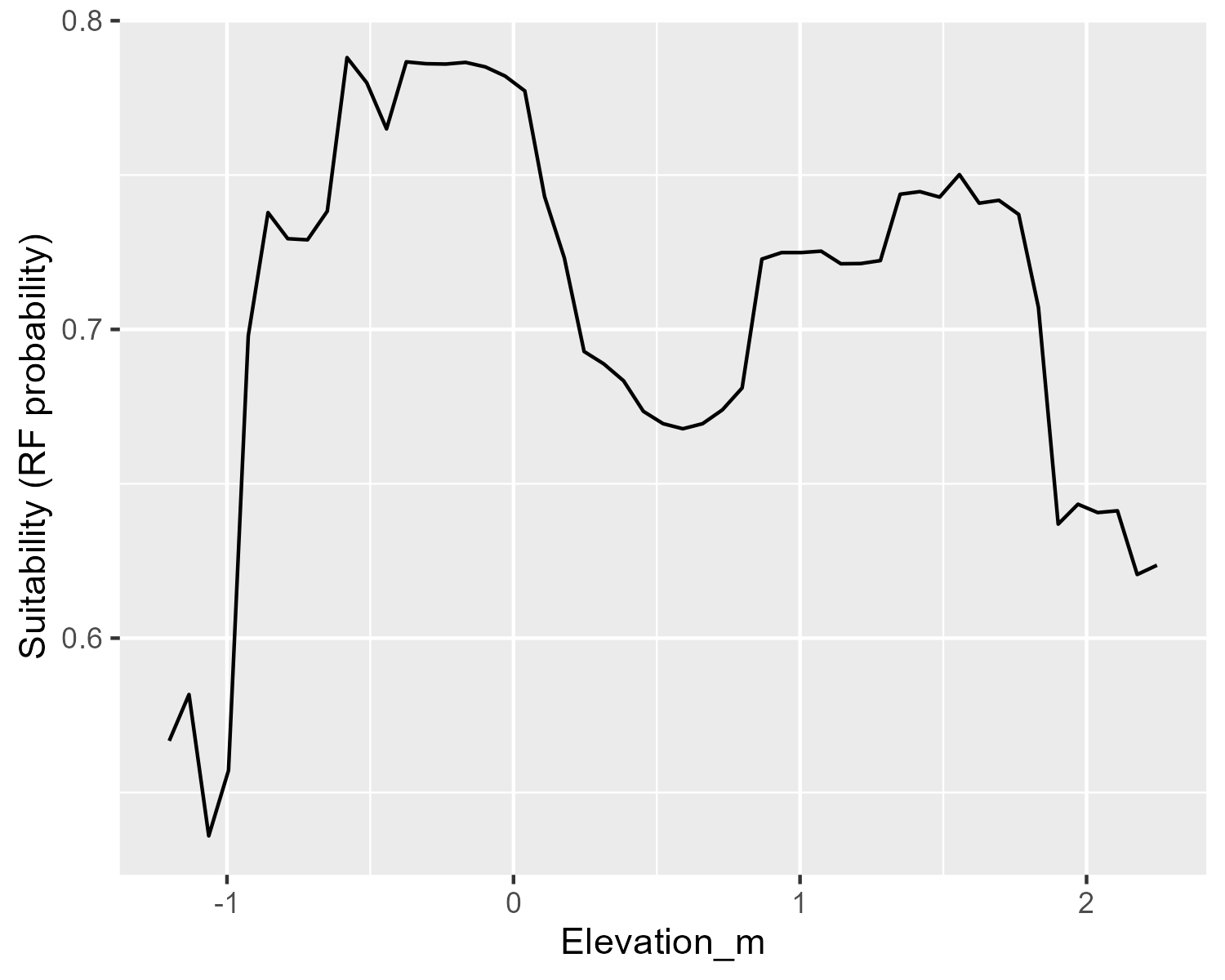


**Fig. S1–S3.** *Partial-dependence (RF probability) for the top three predictors.*


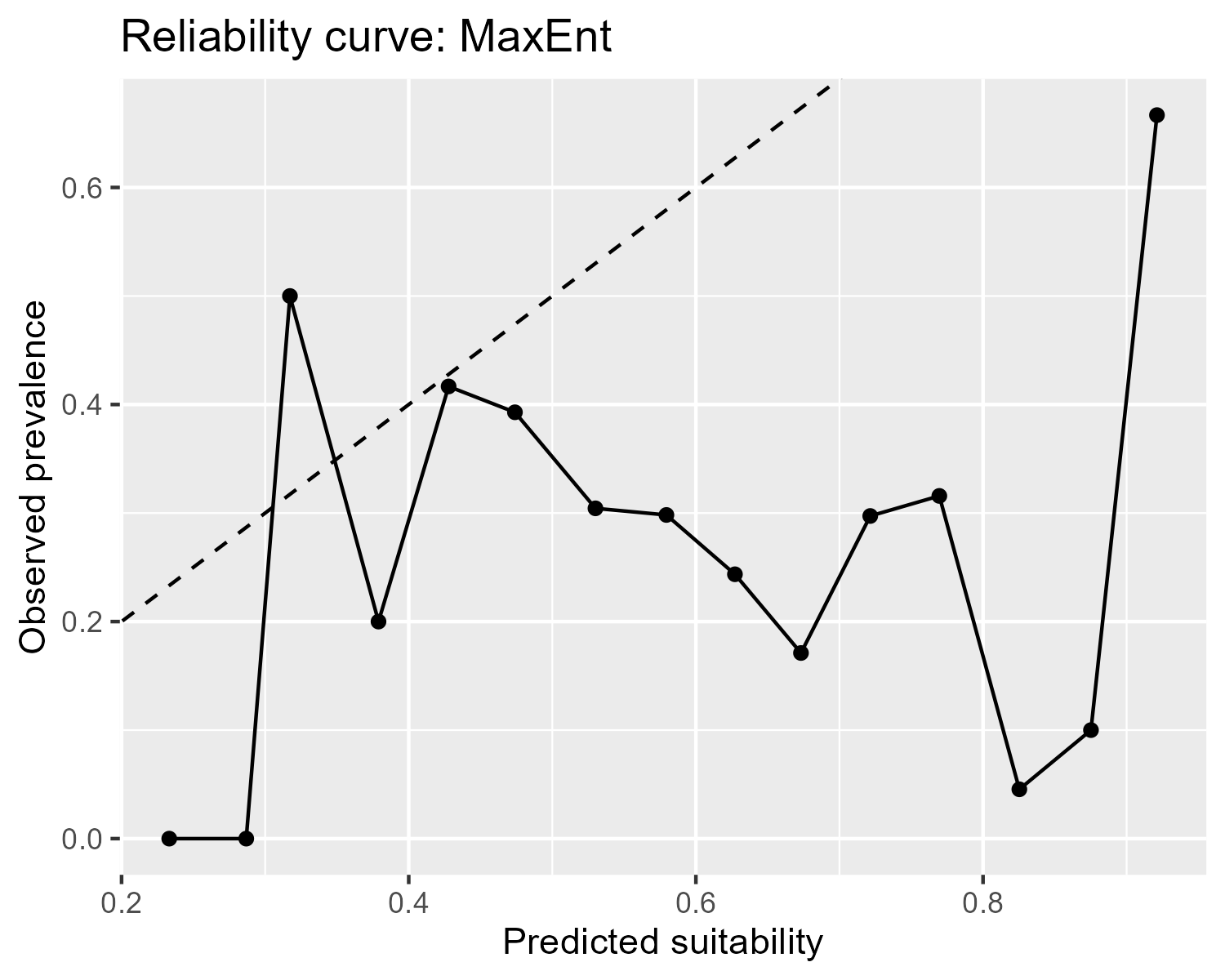


**Fig. S4.** Reliability (calibration) plot and Brier score for the best model.
